# Transcriptome Analysis of Renal Ischemia/Reperfusion Injury and Its Modulation by Ischemic Pre-Conditioning or Hemin Treatment

**DOI:** 10.1371/journal.pone.0049569

**Published:** 2012-11-14

**Authors:** Matheus Correa-Costa, Hátylas Azevedo, Mariane Tami Amano, Giselle Martins Gonçalves, Meire Ioshie Hyane, Marcos Antonio Cenedeze, Paulo Guilherme Renesto, Alvaro Pacheco-Silva, Carlos Alberto Moreira-Filho, Niels Olsen Saraiva Câmara

**Affiliations:** 1 Laboratory of Transplantation Immunobiology, Department of Immunology, Instituto de Ciências Biomédicas, Universidade de São Paulo (USP), São Paulo, Brazil; 2 Laboratory of Pediatric Genomics, Department of Pediatrics, Faculdade de Medicina, Universidade de São Paulo (USP), São Paulo, Brazil; 3 Laboratory of Clinical and Experimental Immunology, Nephrology Division, Universidade Federal de São Paulo (UNIFESP), São Paulo, Brazil; University of Sao Paulo Medical School, Brazil

## Abstract

Ischemia/reperfusion injury (IRI) is a leading cause of acute renal failure. The definition of the molecular mechanisms involved in renal IRI and counter protection promoted by ischemic pre-conditioning (IPC) or Hemin treatment is an important milestone that needs to be accomplished in this research area. We examined, through an oligonucleotide microarray protocol, the renal differential transcriptome profiles of mice submitted to IRI, IPC and Hemin treatment. After identifying the profiles of differentially expressed genes observed for each comparison, we carried out functional enrichment analysis to reveal transcripts putatively involved in potential relevant biological processes and signaling pathways. The most relevant processes found in these comparisons were stress, apoptosis, cell differentiation, angiogenesis, focal adhesion, ECM-receptor interaction, ion transport, angiogenesis, mitosis and cell cycle, inflammatory response, olfactory transduction and regulation of actin cytoskeleton. In addition, the most important overrepresented pathways were MAPK, ErbB, JAK/STAT, Toll and Nod like receptors, Angiotensin II, Arachidonic acid metabolism, Wnt and coagulation cascade. Also, new insights were gained about the underlying protection mechanisms against renal IRI promoted by IPC and Hemin treatment. Venn diagram analysis allowed us to uncover common and exclusively differentially expressed genes between these two protective maneuvers, underscoring potential common and exclusive biological functions regulated in each case. In summary, IPC exclusively regulated the expression of genes belonging to stress, protein modification and apoptosis, highlighting the role of IPC in controlling exacerbated stress response. Treatment with the Hmox1 inducer Hemin, in turn, exclusively regulated the expression of genes associated with cell differentiation, metabolic pathways, cell cycle, mitosis, development, regulation of actin cytoskeleton and arachidonic acid metabolism, suggesting a pleiotropic effect for Hemin. These findings improve the biological understanding of how the kidney behaves after IRI. They also illustrate some possible underlying molecular mechanisms involved in kidney protection observed with IPC or Hemin treatment maneuvers.

## Introduction

Ischemia/reperfusion injury (IRI) is a leading cause of acute renal failure (ARF), a common renal disease that is still associated with high mortality, despite significant advances in the healthcare system [1]. IRI is caused by a sudden transient drop in blood flow associated with a robust inflammatory and oxidative stress response to hypoxia and reperfusion, frequently occurring during shock, sepsis and transplantation [2]. Although important findings have been made in the definition of the cell biologic consequences of IRI [3], [4], there are still few therapies available for this clinical problem [5].

It is known that renal tubular cells response to IRI depends on the intensity and time period of ischemia. Also, many cell phenomena such as proliferation, dedifferentiation, loss of cell polarity and cell death are on tracking during renal IRI [6]. However, the underlying mechanisms participating in the adaptive response occurred along renal IRI need to be clarified in order to understand how to ameliorate the harmful consequences of IRI.

The kidney has the ability to be preconditioned by a non-lethal period of ischemia, which makes it refractory to subsequent ischemia-induced dysfunction in animal models [7]. The ischemic pre-conditioning (IPC) refers to brief episodes of ischemia followed by prolonged ischemia and reperfusion, which protects organs against IRI. This phenomenon can be very useful to understand how kidney uses an endogenous process to protect itself against IRI, revealing whether exogenous influences can mimic this process and, hence, alter the progress of renal acute failure [8].

The kidney can also be protected against IRI by the up-regulation of cytoprotective proteins. For instance, the hyper-expression of the protein heme oxygenase-1 (Hmox1), an isoform of the enzyme involved in the degradation of heme, has shown cytoprotective effects by its end by-products actions as anti-oxidant, anti-inflammatory, anti-apoptotic and anti-proliferative [9]. Indeed, recent studies have highlighted that Hmox1 induction with the drug Hemin is protective in acute and chronic renal insults [10], [11], [12]. Nevertheless, the whole mechanism of action of Hemin is currently poorly understood, and, thus, must be well investigated.

The knowledge of the molecular basis of diseases was facilitated by the advances of high throughput functional genomics, allowing deep analysis of genome-wide results [13]. In this sense, DNA microarray technology can be used to study a complex disease, as it offers the advantage of analyzing thousands of genes simultaneously. This technology coupled with bioinformatics tools may detect changes in genes previously unknown to participate in diseases, identifying possible altered biological functions and providing new drug targets to be investigated. Given this landscape, comparing in what way IPC and Hemin treatment can protect the kidney against IRI could be a relevant approach to analyze which mechanisms are involved in these maneuvers. Based on that, the aim of the present work was to investigate the differential gene expression patterns associated with renal IRI, IPC and Hemin treatment. In addition, gene expression profiles identified for each case were submitted to functional enrichment analysis and a comprehensive literature review was performed to map the state of the art regarding the biological functions overrepresented here.

## Results and Discussion

### Study Design and Analysis

**Figure 1:**
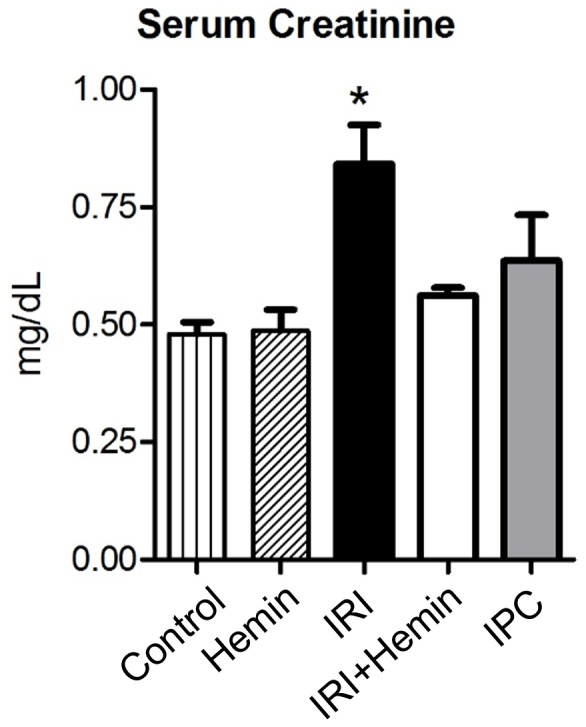
Mean ± standard deviation of serum creatinine concentrations found in mice submitted to different experimental manipulations. C57BL/6 male mice were subjected to surgical and treatment procedures as per described in Materials and Methods. Levels of serum creatinine were measured using the modified Jaffé technique. *Groups were statistically compared using ANOVA followed by Tukey’s post hoc test with p<0.05.

According to surgery and treatment procedures, mice were divided into five groups: Control, IRI, animals pre-treated with Hemin followed by IRI (Hemin + IRI), animals only treated with Hemin (Hemin) and animals pre-conditioned and submitted to IRI (IPC+IRI). Serum creatinine was measured in the groups to verify whether IRI condition has compromised renal function and if IPC and Hemin treatment were able to avoid renal dysfunction (Figure 1). To identify the consequences of IRI, Hemin treatment or IPC in gene expression profile, the following statistical comparisons were made between the groups: IRI *vs* Control, IPC+IRI *vs* Control, IPC+IRI *vs* IRI, IRI+Hemin *vs* IRI and Hemin *vs* Control. These comparisons are described in terms of data and biologically meaningful results obtained hereafter.

### IRI *Versus* Control

**Figure 2:**
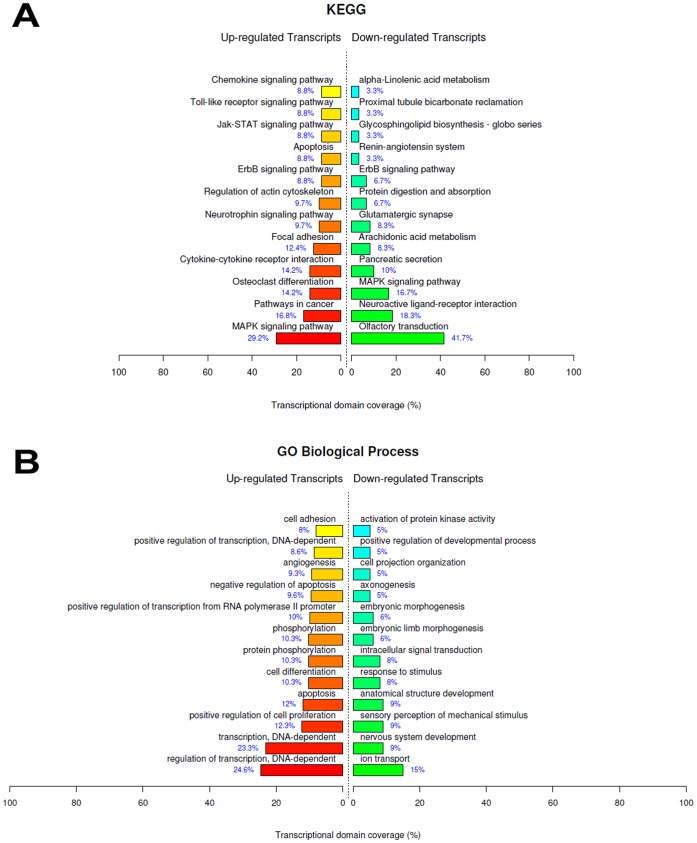
Top ranked biological functions after renal ischemia/reperfusion injury. A) KEGG categories showing significant enriched functions for differentially expressed genes at IRI *vs* Control comparison; B) Biological Process Ontology (GO) of the differentially expressed genes observed at IRI *vs* Control comparison. The bar plot represents the percentage of genes differentially expressed and functionally annotated in isolated kidney tissue. Bar plot colors represent up (red) and down (green) regulated genes.

After statistical testing procedures, removal of the transcripts with no Entrez gene ID and fold change cutoff (Fold ≥3 for up-regulated genes and Fold ≥2 for down-regulated genes), we identified a set of 483 differentially up-regulated and 361 down-regulated genes as a result of IRI. These genes were submitted to functional enrichment analysis through GO (Gene Ontology) and KEGG (Kyoto Encyclopedia Genes and Genomes) databases, and the main biological functions overrepresented are shown in Figure 2. Interestingly, some of the most up-regulated genes in terms of fold change (Table S1 - Cdkn1a, Serpine1, Hmox1, Ccl2 and Plaur) participate of the hypoxia inducible factor-1 (HIF-1) signaling pathway, the master regulator of cellular adaptive responses to hypoxia during IRI, which was shown elsewhere to protect the kidney against this injury through, for example, ischemia-induced angiogenesis [14]. In fact, many genes transcriptionally controlled by HIF and involved in angiogenesis were upregulated after IRI (Table S6– Angiogenesis). Also of note, some genes that are regulated by Protease-Activated Receptor-2 (PAR2) activation such as Fosl1, Serpin1 and Tnfrsf12a are present in Table S1. PAR2 has been implicated with regulation of cytokines and inflammatory condition, in genetic knockout mice studies [15]. During IRI, proteases released from different sources (i.e. inflammatory cells) can activate or up-regulate PAR2. PAR2 agonists have been shown to enhance the efficiency of ischemic preconditioning in the heart [16] and to improve myocardial functional recovery after IRI [17]. Hence, this protein can be a possible relevant target to study in renal ischemic-related diseases, due to its potential protective activity.

### IPC+IRI *Versus* IRI

**Figure 3:**
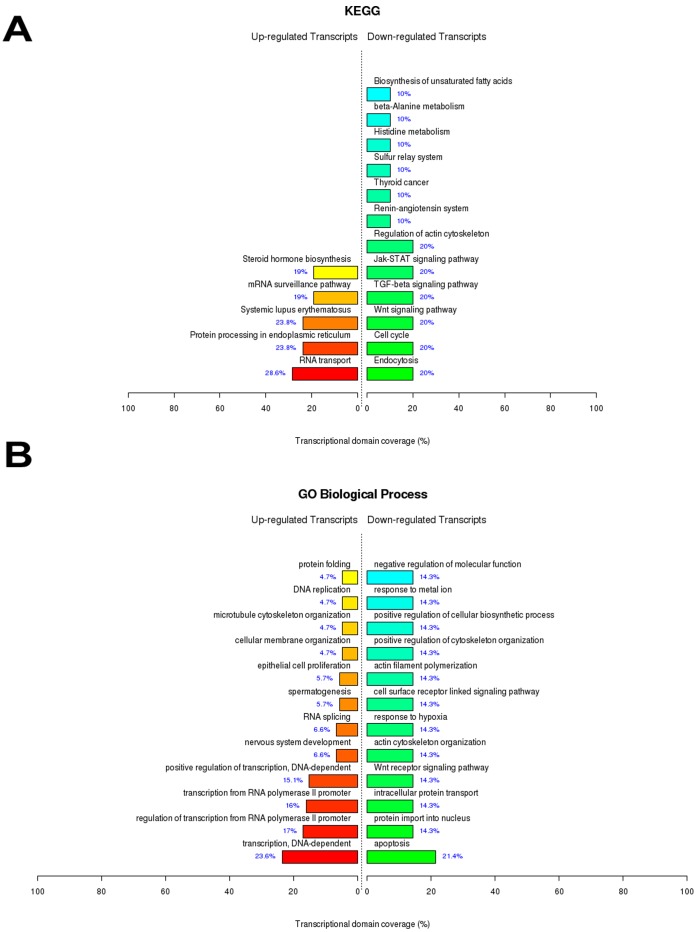
Top ranked biological functions associated with renal ischemic pre-conditioning. A) KEGG categories showing significant enriched functions for differentially expressed genes at IPC+IRI *vs* IRI comparison; B) Biological Process Ontology (GO) of the differentially expressed genes observed at IPC+IRI *vs* IRI comparison. The bar plot represents the percentage of genes differentially expressed and functionally annotated in isolated kidney tissue. Bar plot colors represent up (red) and down (green) regulated genes.

During the preconditioning period of ischemia, many inflammatory trigger substances can be released. These substances bind to membrane receptors and activate intracellular signaling cascades, which, at the end, protect the kidney against IRI. To gain insight into the mechanism of IPC protection in the kidney, we compared the groups IPC and IRI to understand which genes, biological processes and signaling pathways can be possibly involved in it. After statistical testing procedures, removal of the transcripts with no Entrez gene ID and fold change cutoff (genes with fold change ≥3), we found a set of 248 up and 24 down-regulated genes differentially expressed due to IPC. These genes were functionally enriched in significant biological themes, according to Figure 3 or Tables S8 and S9. The most 25 up and 24 down-regulated genes found here are presented in Table S2.

### IPC+IRI *Versus* Control

**Figure 4:**
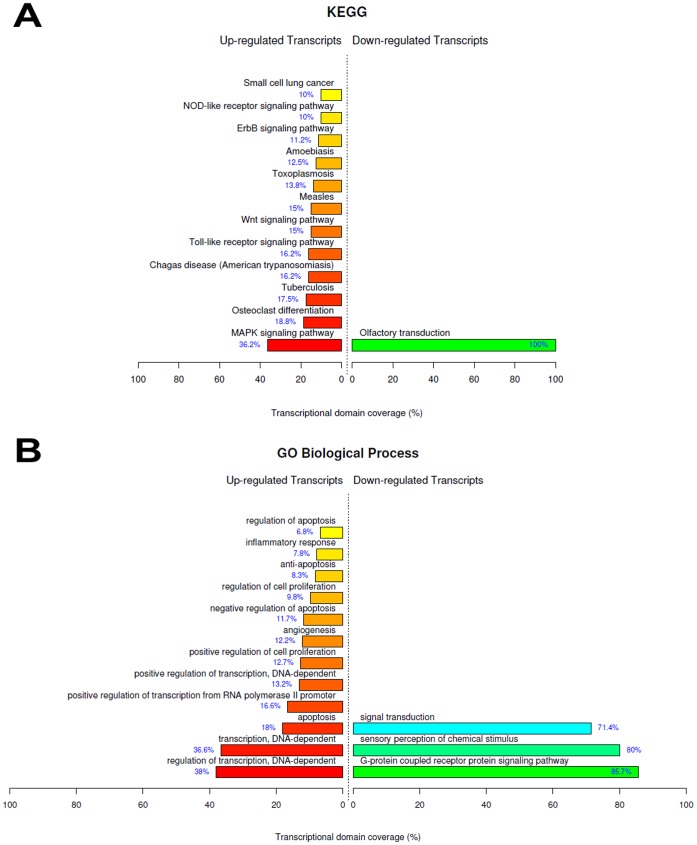
Top ranked biological functions after renal ischemic pre-conditioning and ischemia/reperfusion injury. A) KEGG categories showing significant enriched functions for differentially expressed genes at IPC+IRI *vs* Control comparison; B) Biological Process Ontology (GO) of the differentially expressed genes at IPC+IRI *vs* Control comparison. The bar plot represents the percentage of genes differentially expressed and functionally annotated in isolated kidney tissue. Bar plot colors represent up (red) and down (green) regulated genes.

The comparison between IPC and Control groups was performed to evaluate the gene expression differences between animals submitted to ischemic pre-conditioning followed by IRI and control conditions. After statistical testing procedures, removal of the transcripts with no Entrez gene ID and fold change cutoff (fold change ≥4 for up-regulated genes and fold change ≥2 for down-regulated genes), we found a set of 564 up-regulated genes and 115 down-regulated genes differentially expressed due to IPC+IRI conditions. These genes were functionally enriched in significant biological themes, according to Figure 4 or Tables S10 and S11. It is worth noting that our findings in the renal model of IPC are in accordance with those observed in an IPC model using adult rat hippocampal slice cultures [18]. In both cases, genes pertaining to signaling pathways such as MAPK, Wnt, ErbB and Toll-like receptor were up-regulated. This suggests that possibly the same mechanisms can be observed for IPC in different organs or experimental models. The 25 most up- regulated genes and the 25 most down- regulated genes obtained in this comparison are listed in Table S3.

### IRI + Hemin *Versus* IRI

**Figure 5:**
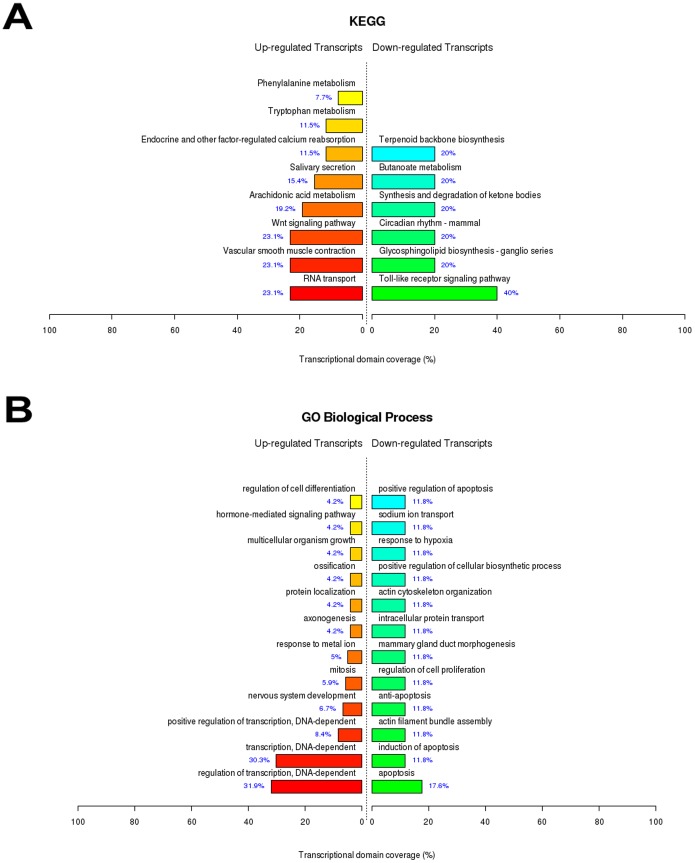
Top ranked biological functions after renal ischemia/reperfusion injury in mice that received previous hemin treatment. A) KEGG categories showing significant enriched functions for differentially expressed genes at IRI+Hemin *vs* IRI comparison; B) Biological Process Ontology (GO) of the differentially expressed genes at IRI+Hemin *vs* IRI comparison. The bar plot represents the percentage of genes differentially expressed and functionally annotated in isolated kidney tissue. Bar plot colors represent up (red) and down (green) regulated genes.

Hemin is capable of reversing renal tubule-interstitial fibrosis [19], a process involved in the renal failure outcome. Although the role of Hemin in the modulation of oxidative stress and inflammation was already described, a broad picture of the molecular mechanisms involved in this protection must be addressed. With this order, IRI+Hemin and IRI groups were compared to establish which genes, pathways and biological processes were modulated by Hemin treatment. After statistical testing procedures and removal of the transcripts with no Entrez gene ID, we identified a set of 306 up-regulated and 29 down-regulated genes as a result of Hemin treatment before IRI. These genes were functionally enriched in some main biological themes, according to Figure 5. For the up-regulated genes set, the most relevant overrepresented biological functions were regulation of transcription, development, mitosis, response to metal ion, regulation of cell differentiation, protein localization, hormone-mediated signaling pathway, RNA transport, vascular smooth muscle contraction, Wnt signaling pathway, arachidonic acid metabolism and amino acid metabolism. For the down-regulated genes set, the most relevant overrepresented biological functions were apoptosis, actin filament bundle assembly, actin cytoskeleton organization, regulation of cell proliferation, protein transport, response to hipoxia, sodium ion transport, toll-like receptor signaling pathway, synthesis and degradation of ketone bodies and circadian rhythm. The 25 most up-regulated genes and the 25 most down-regulated genes obtained in this comparison are posed in the Table S4.

### Hemin *Versus* Control

**Figure 6:**
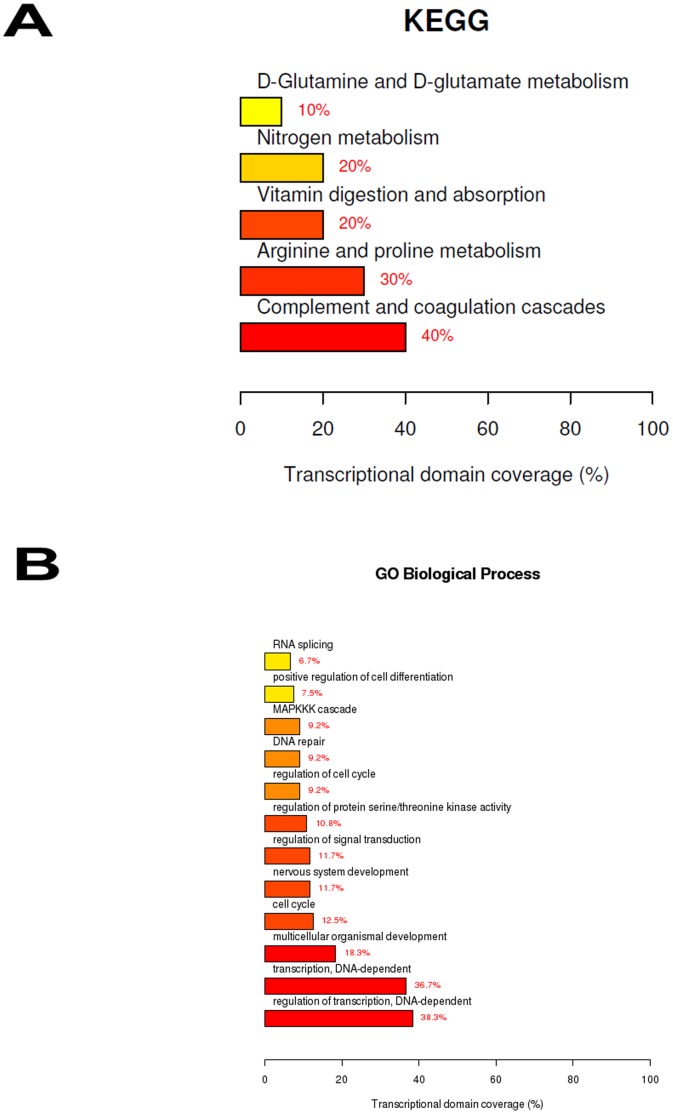
Top ranked biological functions after hemin treatment. A) KEGG categories showing significant enriched functions for differentially expressed genes at Hemin *vs* Control comparison; B) Biological Process Ontology (GO) of the differentially expressed genes at Hemin *vs* Control comparison. The bar plot represents the percentage of genes differentially expressed and functionally annotated in isolated kidney tissue. Bar plot colors represent up (red) and down (green) regulated genes.

The previous induction of Hmox1 in renal tissue is a manner of reducing the subsequent deleterious outcomes of an IRI challenge [20]. Aiming at evaluating the genes, pathways and biological processes involved in this observed better response against IRI, a comparison between Hemin and Control groups was carried out. After statistical testing procedures, removal of the transcripts with no Entrez gene ID and fold change cutoff (genes with fold change ≥3), we found 346 up-regulated genes due to Hemin treatment. These genes were functionally enriched in some main biological themes, according to Figure 6. The most relevant overrepresented biological functions were regulation of transcription, development, cell cycle, regulation of signal transduction, DNA repair, MAPKKK cascade, regulation of cell differentiation, RNA splicing, coagulation cascade and amino acid metabolism. The 25 most up-regulated genes found in this comparison are listed in the Table S5.

### Transcriptome Responses Reveal the Main Biological Functions Modulated by IRI, IPC and Hemin

We discuss hereafter the main cellular phenomena modulated by IRI, IPC and Hemin treatment according to our data. These overrepresented biological functions may explain some protection mechanisms by which IPC and Hemin treatment are able of preserving renal functions after ischemic stress. Also interestingly, mice submitted to IRI showed differential expression of genes belonging not just to harmful but also to putative protective biological functions. This reveals that the transcriptome program induced by ischemia may be also relevant to counter protection against IRI.

#### Stress response

Stress response was overrepresented after IRI and IPC conditions (Tables S6 and S10, respectively), highlighting the relevance of dealing with cell stress under hypoxic conditions. Highly differentially expressed genes observed here after IRI (Table S1) and IPC+IRI (Table S3), such as ATF3 and CHAC1, are expressed in response to eIF2α phosphorylation, a common pathway involved in the response to a variety of stresses. It has been demonstrated that eIF2α phosphorylation controls the commitment to apoptosis during osmotic stress, a kind of stress experienced in IRI [21], [22], and that ATF3 functions as a protector gene against IRI [22]. Furthermore, hypoxia experienced in IRI causes protein synthesis inhibition by activating the unfolded protein response (UPR), an adaptive signaling pathway that promotes cell survival upon accumulation of unfolded proteins in the endoplasmic reticulum (ER). Translation inhibition during this event is caused by eIF2α phosphorylation through its kinase PERK (Protein kinase-like endoplasmic reticulum kinase), present in the ER [23]. In contrast to acute adaptive UPR activation, chronic activation increases expression of proapoptotic mediators, leading to massive apoptosis of kidney cells [24].

#### Apoptosis

We found here that genes belonging to p53 signaling and apoptosis were differentially expressed in IRI, IPC+IRI and IRI+Hemin groups, highlighting the relevance of apoptosis modulation after stress events. Apoptosis of renal tubular epithelial cells plays a major role in acute renal failure [25], since exposure to cellular stress triggers the p53 tumor suppressor factor to induce cell growth arrest or apoptosis. p53 signaling is an important player in the variation of tubular epithelial cells after kidney IRI [26] and p53 inhibitors are capable of protecting kidney against IRI [27], [28]. Moreover, p53 activation mitigates the concomitant activation of protective HIF signaling after IRI, and, thus, the balance between the HIF-1 and p53 responses can determine the IRI outcome [29].

#### MAPK pathway

As depicted in Figure 2, mitogen-activated protein kinase (MAPK) signaling was the most overrepresented theme after IRI. MAPKs mediate the cells response to a wide variety of physiological and stress-related stimuli, including heat shock, ischemia, oxygen free radicals and hyperosmolality, all stresses experienced in IRI. MAPKs activities including JNK, p38 and ERK have been suggested to be markedly enhanced after ischemia *in vivo*. In addition, the relative extent of JNK, p38 or ERK activation has been proposed to determine cell fate after injury (cell survival, necrosis or apoptosis) in post-ischemia/reperfusion [30]. Therefore, MAPK activation can determine renal epithelial cell survival during oxidative injury, and the differences in cellular outcome of the various nephron regions may be related to specific activation of different MAPKs [31]. Also, MAPK pathway intimately participates of the downstream signal transduction associated with innate immune response and subsequent proinflammatory cytokine production. Thus, the balanced regulation of this biochemical pathway is quite relevant to carefully control the inflammation environment created by IRI [32].

The activity of p38 MAPK, JNK and ERK1/2, as well as several enzymes up- and downstream of these pathways, is regulated by their phosphorylation status. Moreover, a complex network of negative regulatory mechanisms present in MAPK signaling prevents deleterious effects of innate immune response. Part of this feedback mechanism is associated with inactivation of MAPKs by phosphatases [33]. Notably, many MAPK signaling phosphatases were found here to be overexpressed after IRI (Table S6– MAPK pathway). The important class of dual specificity phosphatases (DUSP) was remarkably upregulated, showing the relevance of this class of enzymes to regulate MAPK signaling in this context. Also relevant, MAPK signaling was the most up-regulated functionally enriched function observed in the comparison IPC+IRI *vs* Control. Indeed, some studies have already addressed the activation of MAPK signaling by IPC and IRI [34], [35]. Moreover, after Hemin treatment (Hemin x Control), MAPKKK cascade was also one of the overrepresented themes. Taken these multiple lines of evidence together, one can speculate that the attenuation of renal damage effects by MAPKs is an important agenda for future research, which should be fully explored.

#### Innate immune response and JAK/STAT signaling

Trafficking of major innate immunity cellular components into post-ischemic kidneys is stimulated by the expression of several chemokines. For instance, CCL2 and CXCL1 (up-regulated genes, Table S1 and Table S3) respectively attract macrophages and neutrophils to the injury site. Also, the release of pro-inflammatory cytokines such as IL-6, TNF-α and IL-1β mediates innate immune function at the local site of IRI. Many genes encoding for these cytokines were found to be up-regulated in our study, as summarized in the theme “Cytokine-cytokine receptor interaction” (Figure 2). Moreover, the pro-inflammatory JAK/STAT signaling pathway was overrepresented by KEGG analysis (Figure 1). In fact, a study has demonstrated the involvement of JAK/STAT signaling in the pathogenesis of renal IRI, with its blockage resulting in attenuation of renal damage in rats [36]. Therefore, our results suggest that JAK/STAT pathway may also serve as a potential target for early intervention in ischemic acute renal failure.

#### Toll and NOD-like receptors

As observed in Figures 2 and 4, Toll-like (TLRs) and NOD-like receptors (NLRs) signaling pathways were overrepresented after IRI and IPC+IRI. These receptors, which recognize common molecular patterns, participate in the inflammatory response presented in acute and chronic kidney diseases [37], [38], [39]. This activity is mediated by their activation by endogenous ligands released from damaged tissues, such as hyaluronan, fibronectin and heat shock proteins. Also, renal tubular epithelial cells increase the expression of TLRs during acute kidney injury [2]. We found that the TLR-2 expression was altered after IPC and that CD14 expression, a co-receptor for TLR-4 signaling, was up-regulated after IRI and IPC. TLR-4/CD14 signaling was already described as a cellular sentinel for acute renal damage due to the activation of the nuclear factor κB (NF-κB) inflammatory pathway [40], and genes related to I-kappaB kinase/NF-kappaB cascade were up-regulated after IRI (Table S6). This line of evidence suggests that the inflammatory exacerbation promoted by TLR-4/CD14/NF-κB signaling in the kidney may be prejudicial and, thus, these pathways may be a good target to modulate. Moreover, the comparison IRI+Hemin *vs* IRI revealed that TLR signaling pathway was one of the overrepresented down-regulated biological themes (Figure 5). This result highlights the relevance of this signaling in the modulation of IRI by Hemin.

Apart from TLR, the participation of the NLR family in IRI is not well established, although NOD-1 and NOD-2 proteins are expressed in renal tubular epithelial cells after IRI [41]. Furthermore, another study showed that NOD-2 knockout mice presents lower levels of serum creatinine, urea, pro-inflammatory cytokines and chemokines, as well as smaller ischemic tubular necrosis areas compared to wild type animals submitted to IRI [42]. Hence, future studies must be performed to assess the relevance of NLR signaling in IRI and to determine how these receptors are connected with clinical worsening or improvement.

#### Pathways related to differentiation and development

IRI, IPC and Hemin treatment promoted the up-regulation of genes pertaining to cell differentiation and development (Figures 2, 4, 5 and 6; Tables S6, S7, S8, S10, S12 and S14), highlighting their roles in stress-related events. Upon kidney epithelial cells loss due to IRI, the surviving cells dedifferentiate, migrate along the basement membrane, proliferate to restore cell number and then differentiate, restoring the nephron functional integrity [43]. Also interesting, molecules which are expressed during kidney development but not in mature nephron are enormously expressed in proximal tubules after IRI. Although the relevance of the reversion to a less-differentiated cell phenotype during IRI is not clear, such phenomenon must be well understood, given its relevance for the progression to chronic kidney disease.

#### Angiotensin II signaling pathway

The renin-angiotensin-aldosterone system, especially the peptide Angiotensin II (Ang II), contributes to kidney injury [44]. Genes belonging to this biological theme were down-regulated in IRI *vs* Control and IPC+IRI *vs* IRI comparisons (Figures 2 and 3, respectively). In fact, one of the most down-regulated genes in IRI *vs* Control (Table S1) was the receptor of Ang II AT1B. This shows the kidney efforts to down-regulate AT1B expression and, hence, to reduce the renal damage promoted by this receptor signal transduction. Moreover, it is increasingly apparent that the cross-talk between Ang II and other signals such as ErbB through corresponding receptors heterodimerization might play a crucial role in renal deterioration and renal epithelial cell hypertrophy [45], [46]. Therefore, the down-regulation of Ang II receptors may be also a response against this potentially harmful cross-talk.

#### Focal adhesion and ECM-receptor interaction

Focal adhesion and ECM-receptor interaction were other two overrepresented themes observed from the differentially expressed genes found after IRI (Figure 2). The interactions between cell and extracellular matrix are mediated by the integrin family of cell adhesion receptors at focal adhesions, which consist of a large number of both cytoskeletal and signal transduction adapter proteins [47]. Stress events like IRI can disorganize focal adhesions by restructuring actin cytoskeleton and activating some downstream kinases, such as ERK1/2, which causes focal adhesion impairment and subsequent renal injury [48]. Here, we found the up-regulation of genes that participate in focal adhesion and ECM-receptor interaction functions such as CD44, Tenascin and some integrins, which were already described in kidney pathological conditions [49], [50], [51]. As molecules of adhesion, they can be required to avoid massive tubule epithelial cell detachment in response to ischemia, not allowing the disassembly of focal adhesion complexes present in that context. However, they can also promote other functions like cell fate decision and lymphocyte extravasation to the injured local, with the maintenance of a pro-inflammatory milieu within the ischemic kidney [52]. Thus, the preservation of tubular epithelium integrity and focal adhesion interactions that support this epithelium could be an interesting therapeutic approach to maintain renal function stabilized [53].

#### Regulation of actin cytoskeleton

Genes involved in the regulation of actin cytoskeleton were up-regulated at IRI *vs* Control (Figure 2, Table S6) and down-regulated in IRI+Hemin x IRI (Figure 5, Table S13) and IPC+IRI vs IRI comparisons. Actin cytoskeleton plays important roles in the structure and function of proximal tubular epithelial cells through modulating cell-cell and cell-extracellular matrix (ECM) adhesions. Ischemia remodels filamentous actin, detaching proximal tubular epithelial cells, which may lead in part to acute renal failure. Moreover, cellular ATP depletion in diverse cell types results in the net conversion of monomeric G-actin to polymeric F-actin, representing an important aspect of cellular injury in the ischemic tissue. Among the differentially expressed genes found, Rock2 was up-regulated in IRI (Table S6), which participates of actin cytoskeleton polymerization during ischemic conditions. Noteworthy, ROCK-inhibition by hydroxyfasudil was shown to significantly improve kidney function in a rat model of acute renal IRI [54]. Also, we found that Hemin treatment and ischemic pre-conditioning was capable of down-regulating the expression of the gene coding for Profilin (Pfn1). Pfn1 is an actin-binding protein involved in the mechanism of actin polymerization in cellular ATP depletion [55]. Therefore, the regulation of actin polymerization can be an important agenda for future research into the protection against kidney impairment function.

#### Wnt signaling pathway

Wnt signaling pathway genes were differentially regulated in IPC+IRI *vs* IRI (Figure 3), IPC+IRI *vs* Control (Figure 4, Table S10) and IRI +Hemin *vs* IRI (Figure 5, Table S12) comparisons. This found may suggest that Wnt signaling may play a relevant role in the renal protection caused by IPC and Hemin manipulations. Indeed, it is increasingly apparent that Wnt signaling is capable of protecting the kidney against apoptosis by inhibiting Bax, Caspase 3 and Cytochrome c activities, leading, at the end, to ischemic resistance [56], [57]. In contrast, other studies have associated Wnt signaling with the progression of chronic renal allograft damage [58] and renal fibrosis [59]. Perhaps paradoxically, this opposite effect observed may be related to the context-dependency and complexity of this pathway, undergoing many levels of cross-talk with other regulatory pathways. Hence, the predictability of how Wnt pathway modulation could influence renal IRI becomes tricky [60]. Of note, previous studies have addressed the role of Wnt signaling in preconditioning cardioprotection [61], [62], corroborating the idea that some components of Wnt pathway are involved in pro-survival mechanisms elicited by IPC.

#### Vascular events

Genes belonging to “Vascular smooth muscle contraction” category were up-regulated at IRI+Hemin *vs* IRI comparison (Figure 5, Table S12). A previous study has demonstrated that Hmox1 knockout mice exhibited exacerbated vascular lesions after ischemia/reperfusion [63] and their arteries exhibited increased sensitivity to constrictors. Furthermore, it was already demonstrated that hemin treatment improves microcirculation by induction of Hmox1 in IRI after kidney transplantation [64]. Therefore, Hmox1 may be important in the regulation of vessels function in stress situations experienced by the kidney. Hmox1 could neutralize vasoconstrictor mediators released within IRI, counterbalancing detrimental consequences of these mediators to renal function. Also relevant, the IRI+Hemin *vs* IRI comparison showed up-regulation of genes belonging to “Vascular endothelial growth factor receptor signaling pathway” category (Figure 5, Table S12), which may, in turn, help the kidney to avoid persistent ischemia by promoting local angiogenesis.

#### Amino acid and nitrogen metabolism

Hemin treatment enhanced the expression of genes involved in amino acids and nitrogen metabolism (Tables 12 and 14; Figure 6). This finding may indicate a mechanism of tolerance increasing to ischemia by means of a metabolic adaptation through increased amino acid metabolism. Interestingly, the administration of regulatory amino acids such as phenylalanine, tryptophan and alanine showed protective effects against ischemia in a model of hepatic ischemia [65]. Thus, nitrogen metabolism may have a possible role to the preservation of liver viability.

#### Identification of common and exclusively regulated genes between the groups

**Figure 7:**
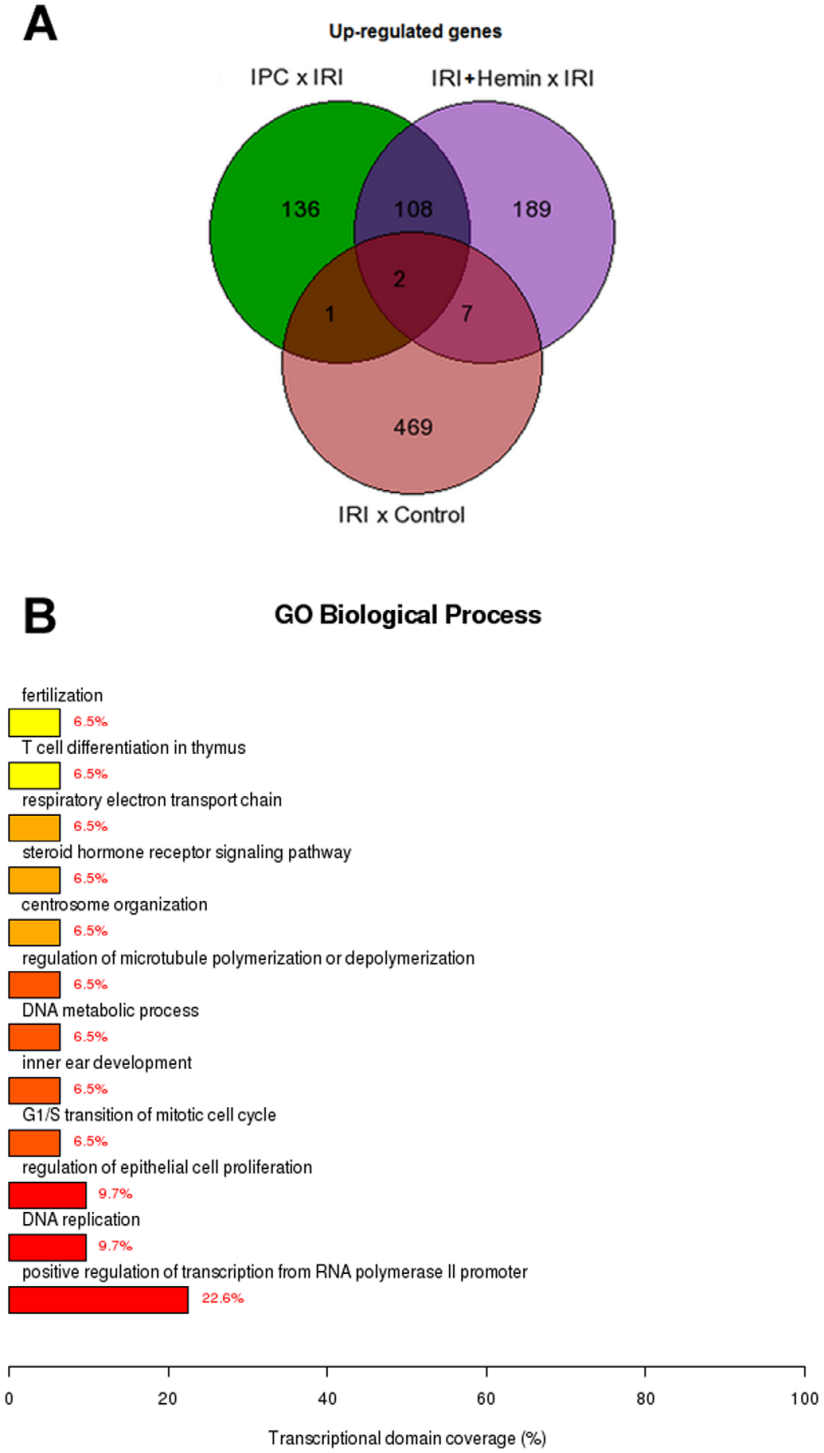
Venn diagram of microarray results reveals similarities and differences in differential transcriptome profiles regulated by IRI, IPC Hemin treatment protocols. A) Venn diagram visualizing the overlapping results between the differentially regulated genes found at IRI vs Control, IRI+Hemin vs IRI and IPC+IRI vs IRI comparisons. B) Biological Process Ontology (GO) of the *commonly* differentially expressed genes found at IPC+IRI *vs* IRI and IRI+Hemin *vs* IRI comparisons. The red color of the bar plots represent up-regulated genes.

**Figure 8:**
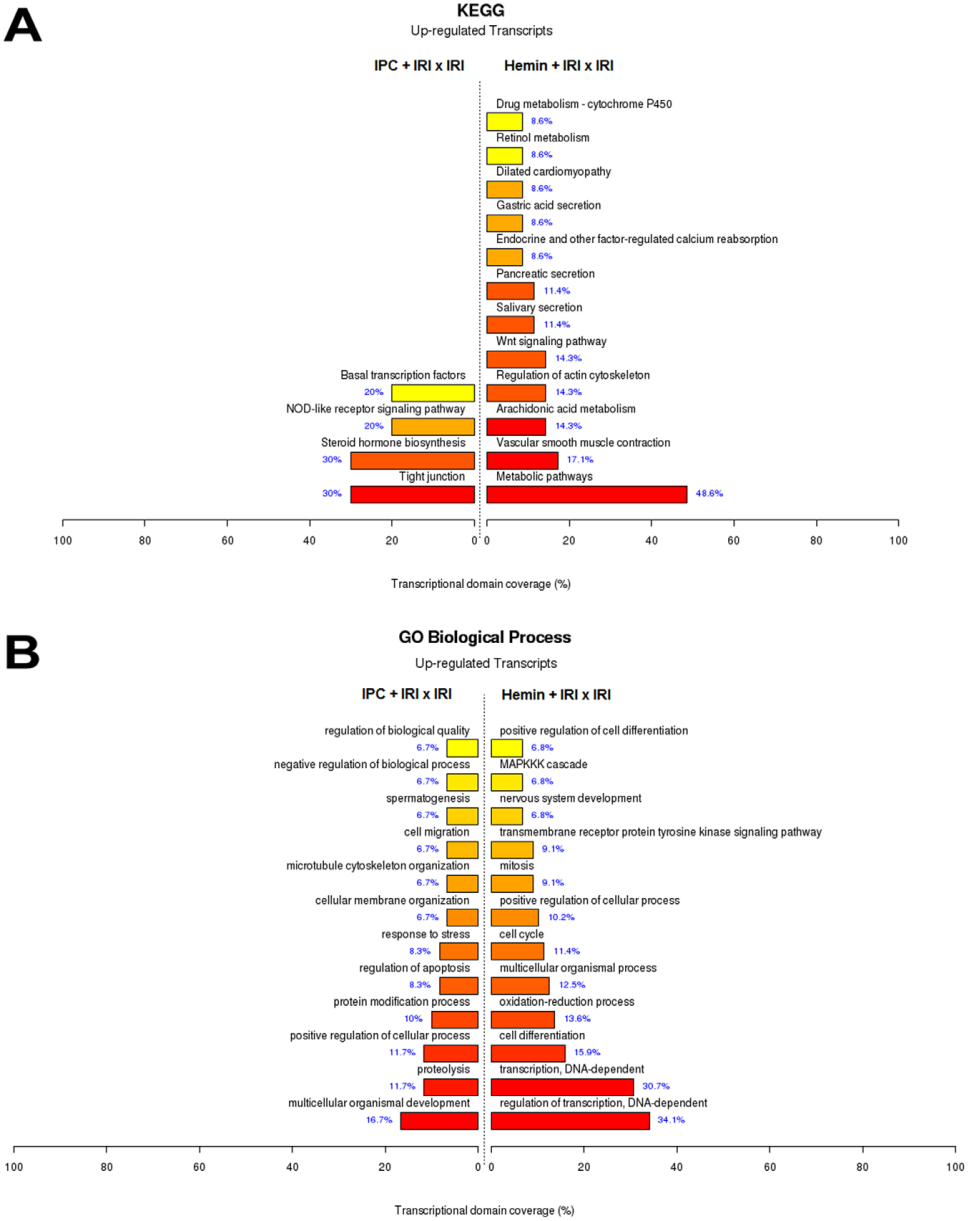
Top ranked biological functions of the *exclusively* differentially expressed genes regulated by IPC and Hemin treatment. A) KEGG categories showing significant functional enrichment of the *exclusively* differentially expressed genes found at IPC+IRI *vs* IRI and IRI+Hemin *vs* IRI comparisons. B) Biological Process Ontology (GO) of the *exclusively* differentially expressed genes found at IPC+IRI vs IRI and IRI+Hemin vs IRI comparisons. The red color of the bar plots represent up-regulated genes.

A Venn diagram was constructed to identify common and exclusively up-regulated genes by IRI, IPC and Hemin treatment protocols (Figure 7A). We found that 136, 189 and 469 genes were exclusively expressed due to IPC, IRI+Hemin or IRI conditions. Although a few genes were commonly up-regulated between IRI *vs* control and the other two comparisons described above, 108 genes were commonly expressed between IPC x IRI and IRI+Hemin x IRI comparisons, indicating possible common mechanisms between IPC and Hemin maneuvers. The main common biological functions enriched were transcription, DNA replication, DNA metabolic process, cell cycle, regulation of microtubule polymerization/depolymerization, centrosome organization and steroid hormone receptor signaling pathway (Figure 7B). In contrast, data representing down-regulated genes did not show a representative amount of commonly differentially expressed genes (data not shown). Figure 8 represents the functional enrichment analysis of the exclusively expressed genes in IPC x IRI and IRI+Hemin x IRI comparisons categorized by KEGG and GO. Noteworthy, IPC exclusively induced the expression of genes involved in tight junction, steroid hormone biosynthesis, NOD-like receptor signaling pathway, proteolysis, response to stress and apoptosis, microtubule cytoskeleton organization and cell migration. On the other hand, Hemin pre-treatment was able to exclusively induce the expression of genes related to metabolic pathways, vascular smooth muscle contraction, arachidonic acid metabolism, regulation of actin cytoskeleton, Wnt signaling pathway, retinol metabolism, Drug metabolism – Cytochrome P450, cell differentiation, oxidation-reduction process, cell cycle, mitosis and MAPK cascade.

#### Validation of microarray results using qRT-PCR

**Figure 9:**
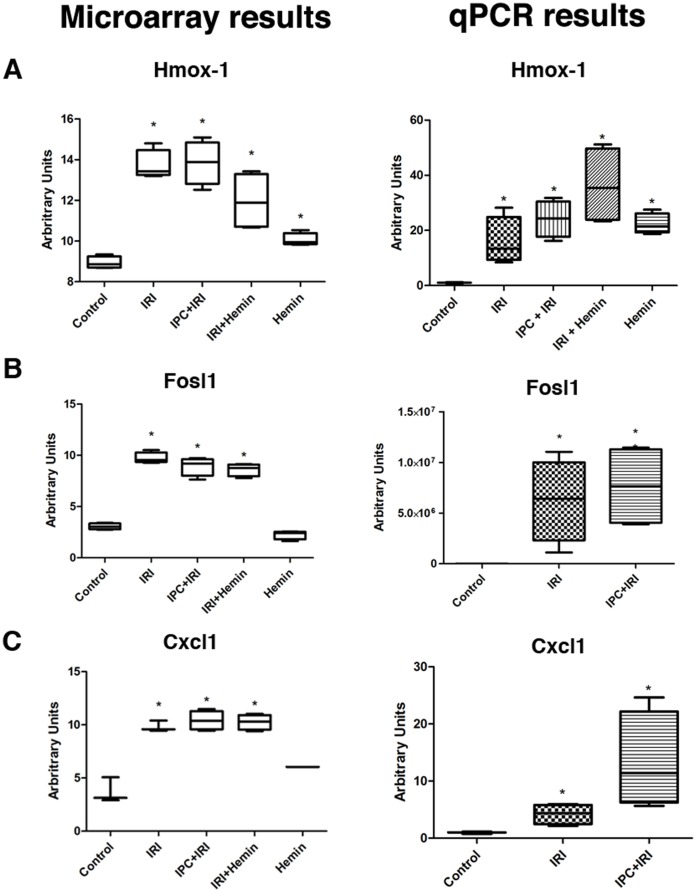
Validation of microarray results by qRT-PCR analysis. Expression of the genes Hmox-1 (A), Fosl1 (B) and Cxcl1 (C), were evaluated by microarray and qRT-PCR experiments. In microarray experiments, values are represented by log2-transformed gProcessed signal. For qRT-PCT, the relative RNA amounts were calculated using the comparative method 2-^ΔΔCT^ and Hprt as an internal control. Results are shown as Boxplot format with whiskers from minimum to maximum values. *p<0.05 compared to Control.

**Figure 10:**
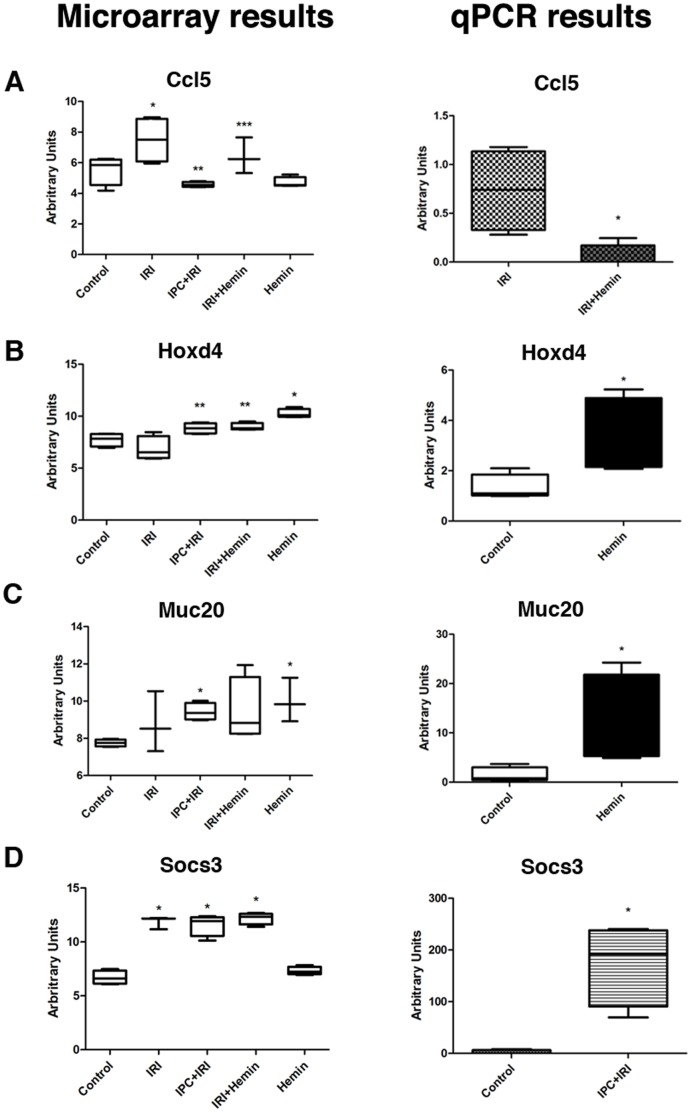
Validation of microarray results by qRT-PCR analysis. Expression of the genes Ccl5 (A), Hoxd4 (B), Muc20 (C) and Socs3 (D) were evaluated by microarray and qRT-PCR experiments. In microarray experiments, values are represented by log2-transformed gProcessed signal. For qRT-PCT, the relative RNA amounts were calculated using the comparative method 2-^ΔΔCT^ and Hprt as an internal control. Results are shown as Boxplot format with whiskers from minimum to maximum values. *p<0.05 compared to Control; **p<0.05 compared to IRI; ***p<0.05 compared to Hemin.

**Figure 11:**
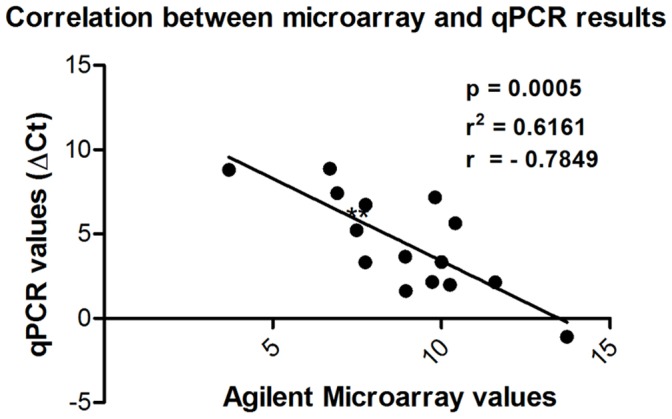
Linear regression analysis revealed a good degree of correlation (R^2^ = 0.6161) between oligonucleotide microarray and quantitative RT-PCR. Horizontal axis, values obtained with microarray experiments; vertical axis, values obtained with qRT-PCR.

Expression patterns of all selected genes (Table S15) used for microarray validation were consistent with the values found using qRT-PCR. Normalization was performed using the Hypoxanthine-guanine phosphoribosyltransferase (Hprt) housekeeping gene. Figures 9, 10 and 11 represent the qRT-PCR results confirming the consistency of microarray data. We found a good correlation between microarray and qPCR results in the genes evaluated (Pearson correlation coefficient, r = −0.7849), which is in accordance with other articles in the literature that showed similar results [66], [67], [68]. Furthermore, the linear regression analysis performed showed a p-value = 0.0005, indicating that the slope of the regression line is significantly different from zero, which in turns shows that there is a significant relationship between “qPCR values” and “DNA microarray values” variables.

### Conclusions

Ischemia/reperfusion injury is still a major clinical problem for many kidney diseases, as well as the predominant insult in organ transplantation. In the present work, we performed transcriptomic analysis of the kidney response to IRI in a mice model of acute kidney injury. Moreover, we compared IRI with two separate interventions, ischemic preconditioning and treatment with Hemin.

Our functional genomics approach revealed in an integrative manner the differential transcriptome profiles and subsequent enriched biological functions among the groups, providing a global picture of how the kidney behaves after IRI. Although previous works have already addressed differential gene expression following IRI in animal models [69], [70], [71], [72], [73], these studies used microarray sets containing much less probes than used here (44000 oligonucleotide probes corresponding to approximately 42000 transcripts). Consequently, many new relevant genes were found in our study, updating the current information regarding transcriptome regulation following IRI. Also, our findings were consistent with these previous microarray studies, confirming the participation of genes associated with apoptosis, inflammation, extracellular matrix, stress response, growth factors, adhesion molecules, proteases, etc. Noteworthy, a consistent part of the differentially expressed genes observed in this work participates in biological functions previously described to be protective against IRI. Therefore, the kidney cells effort to induce a potential protective transcriptome program after IRI was revealed.

Cells injured can release immunological pattern molecules which are recognized by kidney and immunological cells, initiating persistent inflammation. The exacerbation and spreading of inflammatory mediators into other nephron regions may lead to persistent injury of the system. Consonant with this idea, inflammatory soluble mediators such as TGF-β, Angiotensin II and EGF, which signals were described to be altered in our work, may initiate some side effects into kidney. This might affect angiostasis, cell reactions, fibrosis [74] and other pathophysiological processes such as epithelial–mesenchymal transition process, in which injured tubular cells trying to regenerate the renal system recapitulate its development [75]. Accordingly, studies aiming to tune these pathways may clarify in which conditions these signals may cause an exacerbated damage response, depending on factors such as time, site of release and specific mediators.

Hmox1 up-regulation was already described as a common mechanism of protection afforded by Hemin and IPC in other experimental models [64], [76], [77], [78]. Hemin treatment is widely known to induce Hmox-1 expression [11], [19], [79], [80], [81]. Moreover, some recent papers have shown that, in different organs, the protective effects of IPC are at least partially mediated via Hemox-1 up regulation [82], [83], [84], [85]. In addition, some studies showed that the inhibition of Hmox-1 (either by pharmacologic inhibition or with siRNA) causes the lost of the protective effects of IPC [76], [86], confirming once more the role of heme oxygenase-1 system in tissue recovery.

Beside the previous described mechanism, new insights were also obtained about the underlying mechanisms involved in kidney protection against IRI promoted by IPC and Hemin treatment. Venn diagram analysis helped us to identify overlapping differentially expressed genes, which may suggests, in turn, potential common and different mechanisms involved in these maneuvers (Figures 7 and 8). Both maneuvers were able to modulate genes belonging to DNA replication, proliferation, cell cycle, microtubule polymerization and centrosome organization. Collectively, these results suggest that the up regulation of pathways that facilitate cell recovery is occurring within kidney tissue in both cases. Accordingly, one of the main pathways overexpressed after Hemin treatment and IPC is the MAPK signaling, which is involved in cell cycle division and, consequently, proliferation. As an example of this cascade, a recent paper showed that when there is a pharmacological intervention that leads to inhibition of MAPK signaling, the recovery after an acute kidney injury episode is delayed [87].

Venn diagram analysis also showed us some possible exclusive mechanisms between Hemin and IPC. IPC exclusively regulated the expression of genes pertaining to protein modification processes such as proteolysis, response to stress and regulation of apoptosis. Therefore, the role of IPC in controlling exacerbated stress response and consequently protein modifications and apoptosis was highlighted. Finally, Hemin treatment exclusively regulated the expression of genes associated with cell differentiation, metabolic pathways, cell cycle, mitosis, development, regulation of actin cytoskeleton and arachidonic acid metabolism, suggesting a pleiotropic effect for Hemin.

We only tested in this work gene expression changes at 6 hours interval. Thus, further studies to evaluate temporal gene expression of selected genes with biological relevance should be carried out to confirm which changes are transient or durable. We found relevant this time interval because unpublished data from our laboratory showed that the peak of Hmox-1 expression occurs around 6 hours after reperfusion, providing a reasonable evidence that this time point should be the best to compare changes between the groups shown in our manuscript. As we focused on the transcriptome analysis, we believe that the chosen time point may provide us essential information upon the molecular changes on gene expression that will later interfere in the renal function outcome, as observed by our group and others [10], [88], [89]. Also, this temporal gene expression analysis could clarify some issues, as many results in literature reveal opposite roles for the same signals, with the duration of specific pathways shaping the switch between one and another response [90].

Although the functional enrichment analysis used here was helpful to identify possible relevant genes and pathways, a limitation of the present study is that we were only able to detect pathways whose genes are regulated by transcriptional activity. However, other relevant targets that are not regulated at the transcriptional level may have not been disclosed. These targets could be possibly regulated metabolically, dependent on phosphorylation status or other indirect effects of IPC or hemin treatment.

In summary, the functional transcriptional analysis conducted in this work allowed the detection of new targets, biological processes and signaling pathways associated with IRI and renoprotective defenses. Further dissections of the molecular mechanisms found here are demanding to gain potential insights into the pathophysiological changes occurring in renal IRI. Moreover, further studies evaluating the protection properties of IPC and Hemin treatment may be an important agenda for the discovery of effective treatments to ischemia-related kidney diseases.

## Materials and Methods

### Animal and Surgical Procedures

Isogenic male C57BL/6 mice, age 8–12 wks (25–28 g), were purchased from Federal University of São Paulo (UNIFESP), Brazil. All animals were housed in individual and standard cages and had free access to water and food. All procedures were previously reviewed and approved by the internal Ethics Committee of the Institution (document number 118/2008).

### Experimental Model of Renal IRI

Surgery was performed as previously described [91]. Briefly, mice were anesthetized with Ketamine-Xylazine (Agribrands do Brazil, São Paulo, Brazil), a midline incision was made and both renal pedicles were cross-clamped for 45 minutes. During the procedure, animals were kept well hydrated with saline and at a constant temperature (∼37°C) through a heating pad device. Subsequently, microsurgery clamps were removed, the abdomen closed and animals placed in single cages, warmed by indirect light until completely recovered from anesthesia. Animals were kept under adjustable conditions until sacrifice, namely 6 hours after renal reperfusion. In order to induce Hmox1 expression, mice received Hemin (Frontier Scientific, Canada) i.p., 25 mg/kg, 24 hours prior to surgery. For ischemic preconditioning protocol, mice were anesthetized and both renal pedicles were clamped for 15 minutes. After that, microsurgery clamps were removed and animals were sutured. Mice were kept under adjustable conditions for 7 days, and then they underwent standard renal IRI model (45 minutes of ischemia and sacrifice after 6 hours of reperfusion).

### Analysis of Renal Function

Serum creatinine was used for evaluation of renal function. Blood samples were collected at 6 hours post reperfusion from the abdominal inferior cava vein immediately before induced death. Serum samples were analyzed on a Cobas Mira Plus (Roche, Mannhein, Germany), using the modified Jaffé technique. Groups were statistically compared using one-way ANOVA followed by Tukey’s post-hoc test with p<0.05.

### RNA Extraction

Total RNA was isolated from kidney samples using Trizol reagent (Life Technologies) and purified using RNeasy Spin Columns (Qiagen, USA). RNA quantity was determined using a Nanovue spectrophotometer (GE Healthcare, USA). The RNA quality was performed using a 2100 Bioanalyzer with an RNA 6000 Nano kit and Ladder (Agilent Technologies, USA), according to the manufacturer’s instructions. The Bioanalyzer produces an electropherogram, which shows the distribution of RNA transcripts in the sample. In an ideal sample, the two peaks of the ribosomal RNA 18S and 28S bands are observed, while additional peaks suggest RNA degradation and/or DNA contamination. The 2100 Bioanalyzer Expert software program (version B.02.06.SI418) assigns an RNA integrity number (RIN) from 1 to 10, with 1 = degraded and 10 = intact (Schroeder et al, 2006). Only samples with a RNA integrity number (RIN) of 8 or greater were employed.

### RNA Amplification and Labeling

Agilent’s Quick Amp Labeling Kit was used to generate fluorescent cRNA (complementary RNA) for the microarray hybridizations, following the manufacturer’s instructions. Briefly, a 700 ng aliquot of total RNA was reverse transcribed into cDNA. Synthesized cDNA was transcribed into cRNA and labeled with the fluorescent dye Cyanine 3 (Cy3). Labeled cRNA was purified with RNeasy Mini columns (Qiagen). The quality of each cRNA sample was verified by total yield and Cy3 specific activity calculated based on Nanovue spectrophotometer measurements (GE Healthcare).

### Microarray Hybridization

Microarray hybridizations were carried out on labeled cRNAs with Cy3 specific activity greater than 9 ρmol Cy3 per µg of RNA. Arrays were incubated at 65°C for 17 h in Agilent’s microarray hybridization chambers and subsequently washed according to Agilent’s one-color microarray-based gene expression analysis protocol (Version 5.7, March 2008). Gene expression profiles were evaluated using Agilent whole mouse genome 4×44 K oligonucleotide microarrays.

### Data Acquisition

Hybridized slides were scanned at 5 µm resolution using an Agilent G2505B DNA microarray scanner. Default settings were modified to scan the same slide twice at two different sensitivity levels (XDR Hi 100% and XDR Lo 10%). The two linked images generated were analyzed together and data was extracted subtracting the background using the standard procedures contained in the Agilent Feature Extraction (FE) Software version 9.5.1. The software returns a series of spot quality measures to assess the reproducibility and reliability of spot intensity estimates. These parameters are summarized in a quality control report and were evaluated in order to support the high quality of the data acquired.

### Data Processing and Analysis

The R statistical environment (http://www.r-project.org) was used to filter the data. The mean of the probes for each gene was calculated, genes with more than one missing value were removed and then the signal intensities were log2 transformed. These logarithmic values were input in the TM4 software suite in order to perform the statistical analyses [92]. To identify which genes were significantly differentially expressed, a statistical technique called SAM (Significance Analysis of Microarrays) was used [93]. SAM assigns a score to each gene on the basis of a change in gene expression relative to the standard deviation of repeated measurements. For genes with scores greater than an adjustable threshold, SAM uses permutations of the repeated measurements to estimate the percentage of genes identified by chance – the false discovery rate (FDR). Analysis parameters (Delta) were set to result in zero FDR. Fold changes based on arbitrary selection criteria (3 or 4-fold change up or 2-fold change down) were applied to identify only the genes whose expression was most affected during the different protocols used here. Combinations of gene sets were carried out using Gene List Venn Diagrams software [94]. The complete data set is publicly available at the National Center for Biotechnology Information (NCBI) Gene Expression Omnibus (http://www.ncbi.nlm.nih.gov/geo/) through the series accession number GSE39548.

### Functional Enrichment Analysis

Overrepresented biological functions were searched in the differential gene expression datasets using FunNet (http://www.funnet.info). FunNet is an integrative functional genomics tool that performs a functional profiling of gene expression data, identifying overrepresented biological themes [95]. FunNet exploits genomic annotations provided by Gene Ontology (GO) and Kyoto Encyclopedia of Genes and Genomes (KEGG) databases [96], [97].

### Validation of Microarray Results by Real-time PCR (qPCR)

To validate the results of microarray analysis, we confirmed by qPCR the differential expression of some representative genes using the same RNA samples that were used for microarray. First-strand cDNA was synthesized from DNAse-treated total RNA using Moloney Murine Leukemia Virus Reverse Transcriptase (Promega, Wisconsin, US). We used Taqman-based quantitative RT-PCR for validation of some of the genes identified by the microarray analysis. The comparative method 2^−ΔΔCT^ was used for relative quantification of gene transcription between the studied groups [98] and gene expression levels were normalized to *Hprt* gene. Predesigned Taqman primers (Life Technologies) were used for the validation. The list of genes validated by qRT-PCT with the corresponding probe numbers are presented in Table S15. DNA microarray and qRT-PCR results were subjected to linear regression analysis and the Pearson coefficient correlation was calculated to verify the degree of correlation between these two methodologies using GraphPad Prism® 5. ANOVA one way with Tukey’s *post hoc* test or Student’s t-test were performed to verify significant differences between the groups in microarray and qRT-PCR results. For all tests, statistical significance was established as a *P* value of <0.05.

## Supporting Information

Table S1 **Gene profile comparison between IRI and control groups.**
(DOC)

Table S2 **Gene profile comparison between IPC and IRI groups.**
(DOC)

Table S3 **Gene profile comparison between IPC and control groups.**
(DOC)

Table S4 **Gene profile comparison between Hemin+IRI and IRI groups.**
(DOC)

Table S5 **Gene profile comparison between Hemin and Control groups.**
(DOC)

Table S6 **Up regulated genes in IRI group (vs control), according to GO and KEGG categories.**
(DOC)

Table S7 **Down regulated genes in IRI group (vs control), according to GO and KEGG categories.**
(DOC)

Table S8 **Up regulated genes in IPC group (vs IRI), according to GO and KEGG categories.**
(DOC)

Table S9 **Down regulated genes in IPC group (vs IRI), according to GO and KEGG categories.**
(DOC)

Table S10 **Up regulated genes in IPC group (vs control), according to GO and KEGG categories.**
(DOC)

Table S11 **Down regulated genes in IPC group (vs control), according to GO and KEGG categories.**
(DOC)

Table S12 **Up regulated genes in Hemin+IRI group (vs IRI), according to GO and KEGG categories.**
(DOC)

Table S13 **Down regulated genes in Hemin+IRI group (vs IRI), according to GO and KEGG categories.**
(DOC)

Table S14 **Up regulated genes in Hemin group (vs control), according to GO and KEGG categories.**
(DOC)

Table S15 **List of selected genes used for microarray results validation by qRT-PCR.**
(DOC)
