# Supplementary material for: Transcriptome Analysis of Renal Ischemia/Reperfusion Injury and Its Modulation by Ischemic Pre-Conditioning or Hemin Treatment
Source: PLoS One. 2012 Nov 14;7(11):e49569. doi: 10.1371/journal.pone.0049569 (PMC3498198; doi:10.1371/journal.pone.0049569)
Supplement: Table S1 — Gene profile comparison between IRI and control groups. (DOC) [file pone.0049569.s001.doc]

**Table S1**. Gene profile comparison between IRI and control groups.

| | **Name** | **Symbol** | **Fold change** | | | | --- | --- | --- | --- | --- | | fos-like antigen 1 | Fosl1 | | 101.8 |  | | chemokine (C-X-C motif) ligand 1 | Cxcl1 | | 69.0 |  | | v-maf musculoaponeurotic fibrosarcoma oncogene family, protein F (avian) | Maff | | 68.8 |  | | activating transcription factor 3 | Atf3 | | 51.8 |  | | ChaC, cation transport regulator-like 1 (E. coli) | Chac1 | | 44.6 |  | | SRY-box containing gene 9 | Sox9 | | 41.3 |  | | cyclin-dependent kinase inhibitor 1A (P21) | Cdkn1a | | 40.0 |  | | cholesterol 25-hydroxylase | Ch25h | | 36.7 |  | | suppressor of cytokine signaling 3 | Socs3 | | 35.8 |  | | lipocalin 2 | Lcn2 | | 31.3 |  | | chemokine (C-C motif) ligand 2 | Ccl2 | | 31.0 |  | | serine (or cysteine) peptidase inhibitor, clade E, member 1 | Serpine1 | | 30.3 |  | | plasminogen activator, urokinase receptor | Plaur | | 30.2 |  | | heme oxygenase (decycling) 1 | Hmox1 | | 27.6 |  | | tumor necrosis factor receptor superfamily, member 12a | Tnfrsf12a | | 25.6 |  | | sphingosine kinase 1 | Sphk1 | | 24.7 |  | | absent in melanoma 1-like | Aim1l | | 24.1 |  | | similar to gag protein | LOC100047599 | | 22.9 |  | | inhibin beta-B | Inhbb | | 20.8 |  | | Eph receptor A2 | Epha2 | | 20.1 |  | | FBJ osteosarcoma oncogene B | Fosb | | 19.4 |  | | tribbles homolog 3 (Drosophila) | Trib3 | | 18.3 |  | | myelocytomatosis oncogene | Myc | | 18.2 |  | | S100 calcium binding protein A8 (calgranulin A) | S100a8 | | 18.0 |  | | FBJ osteosarcoma oncogene | Fos | | 17.7 |  | | WD repeat domain 86 | Wdr86 | | -14.6 |  | | 2-hydroxyacyl-CoA lyase 1 | Hacl1 | | -9.3 |  | | ciliary rootlet coiled-coil, rootletin | Crocc | | -9.0 |  | | solute carrier family 4 (anion exchanger), member 4 | Slc4a4 | | -8.3 |  | | RIKEN cDNA D630023F18 gene | D630023F18Rik | | -7.5 |  | | RIKEN cDNA D630039A03 gene | D630039A03Rik | | -6.9 |  | | RIKEN cDNA D130020G16 gene | D130020G16Rik | | -6.7 |  | | angiotensin II receptor, type 1b | Agtr1b | | -6.5 |  | | SLIT and NTRK-like family, member 6 | Slitrk6 | | -6.2 |  | | transient receptor potential cation channel, subfamily C, member 3 | Trpc3 | | -6.2 |  | | olfactory receptor 620 | Olfr620 | | -6.2 |  | | T cell leukemia, homeobox 2 | Tlx2 | | -6.2 |  | | KN motif and ankyrin repeat domains 4 | Kank4 | | -6.0 |  | | RIKEN cDNA 9530096D07 gene | 9530096D07Rik | | -5.7 |  | | TLR4 interactor with leucine-rich repeats | Tril | | -5.7 |  | | matrix metallopeptidase 28 (epilysin) | Mmp28 | | -5.7 |  | | K+ voltage-gated channel, subfamily S, 2 | Kcns2 | | -5.6 |  | | zinc finger protein 862 | Zfp862 | | -5.6 |  | | carbohydrate sulfotransferase 12 | Chst12 | | -5.5 |  | | thromboxane A2 receptor | Tbxa2r | | -5.5 |  | | EMI domain containing 2 | Emid2 | | -5.4 |  | | zinc finger protein 750 | Zfp750 | | -5.4 |  | | thyrotroph embryonic factor | Tef | | -5.4 |  | | apelin receptor | Aplnr | | -5.4 |  | | Bardet-Biedl syndrome 10 (human) | Bbs10 | | -5.4 |  | |  |  |
| --- | --- | --- | --- | --- | --- | --- | --- | --- | --- | --- | --- | --- | --- | --- | --- | --- | --- | --- | --- | --- | --- | --- | --- | --- | --- | --- | --- | --- | --- | --- | --- | --- | --- | --- | --- | --- | --- | --- | --- | --- | --- | --- | --- | --- | --- | --- | --- | --- | --- | --- | --- | --- | --- | --- | --- | --- | --- | --- | --- | --- | --- | --- | --- | --- | --- | --- | --- | --- | --- | --- | --- | --- | --- | --- | --- | --- | --- | --- | --- | --- | --- | --- | --- | --- | --- | --- | --- | --- | --- | --- | --- | --- | --- | --- | --- | --- | --- | --- | --- | --- | --- | --- | --- | --- | --- | --- | --- | --- | --- | --- | --- | --- | --- | --- | --- | --- | --- | --- | --- | --- | --- | --- | --- | --- | --- | --- | --- | --- | --- | --- | --- | --- | --- | --- | --- | --- | --- | --- | --- | --- | --- | --- | --- | --- | --- | --- | --- | --- | --- | --- | --- | --- | --- | --- | --- | --- | --- | --- | --- | --- | --- | --- | --- | --- | --- | --- | --- | --- | --- | --- | --- | --- | --- | --- | --- | --- | --- | --- | --- | --- | --- | --- | --- | --- | --- | --- | --- | --- | --- | --- | --- | --- | --- | --- | --- | --- | --- | --- | --- | --- | --- | --- | --- | --- | --- | --- | --- | --- | --- | --- | --- | --- | --- | --- | --- | --- | --- | --- | --- | --- | --- | --- | --- | --- | --- | --- | --- | --- | --- | --- | --- | --- | --- | --- | --- | --- | --- | --- | --- | --- | --- | --- | --- | --- | --- | --- | --- | --- | --- | --- | --- | --- | --- | --- | --- | --- | --- |

Most 25 up and 25 down regulated genes found in the kidney tissue after ischemia/reperfusion injury (IRI *vs* Control). Gene expression fold changes are represented by IRI group gene expression values in relation to Control values.
