# Supplementary material for: Transcriptome Analysis of Renal Ischemia/Reperfusion Injury and Its Modulation by Ischemic Pre-Conditioning or Hemin Treatment
Source: PLoS One. 2012 Nov 14;7(11):e49569. doi: 10.1371/journal.pone.0049569 (PMC3498198; doi:10.1371/journal.pone.0049569)
Supplement: Table S2 — Gene profile comparison between IPC and IRI groups. (DOC) [file pone.0049569.s002.doc]

**Table S2**. Gene profile comparison between IPC and IRI groups.

| **Name** | **Symbol** | **Fold change** |
| --- | --- | --- |
| Rn28s1 28S ribosomal RNA | Rn28s1 | 35.1 |
| Rn18s 18S ribosomal RNA | Rn18s | 28.4 |
| solute carrier family 7 (cationic amino acid transporter, y+ system), member 12 | Slc7a12 | 15.7 |
| StAR-related lipid transfer (START) domain containing 4 | Stard4 | 10.9 |
| ring finger protein 214 | Rnf214 | 9.6 |
| heat shock protein 4 | Hspa4 | 9.0 |
| membrane magnesium transporter 2 | Mmgt2 | 8.5 |
| thyrotroph embryonic factor | Tef | 8.1 |
| 2-hydroxyacyl-CoA lyase 1 | Hacl1 | 7.6 |
| RIKEN cDNA 5730416O20 gene | 5730416O20Rik | 7.4 |
| fatty acid binding protein 7, brain | Fabp7 | 7.4 |
| apoptotic chromatin condensation inducer 1 | Acin1 | 7.4 |
| leucine zipper protein 1 | Luzp1 | 7.3 |
| cytoskeleton-associated protein 4 | Ckap4 | 7.2 |
| RIKEN cDNA 4931406H21 gene | 4931406H21Rik | 6.9 |
| kelch-like 15 (Drosophila) | Klhl15 | 6.8 |
| H1 histone family, member X | H1fx | 6.8 |
| golgi autoantigen, golgin subfamily b, macrogolgin 1 | Golgb1 | 6.5 |
| synaptotagmin X | Syt10 | 6.3 |
| family with sequence similarity 168, member A | Fam168a | 6.3 |
| nischarin | Nisch | 6.3 |
| histone cluster 3, H2ba | Hist3h2ba | 6.2 |
| TAF1 RNA polymerase II, TATA box binding protein (TBP)-associated factor | Taf1 | 6.0 |
| ELOVL family member 7, elongation of long chain fatty acids (yeast) | Elovl7 | 5.9 |
| DEAD (Asp-Glu-Ala-Asp) box polypeptide 25 | Ddx25 | 5.9 |
| solute carrier family 4, sodium bicarbonate cotransporter, member 7 | Slc4a7 | -33.5 |
| superoxide dismutase 3, extracellular | Sod3 | -8.9 |
| formin-like 1 | Fmnl1 | -7.1 |
| myelocytomatosis oncogene | Myc | -6.2 |
| E1A binding protein p300 | Ep300 | -6.0 |
| karyopherin (importin) alpha 4 | Kpna4 | -6.0 |
| profilin 1 | Pfn1 | -4.8 |
| carnosine dipeptidase 1 (metallopeptidase M20 family) | Cndp1 | -3.7 |
| RIKEN cDNA C030046I01 gene | C030046I01Rik | -3.1 |
| ribonuclease, RNase K | Rnasek | -2.7 |
| biliverdin reductase A | Blvra | -2.7 |
| mitogen-activated protein kinase 4 | Mapk4 | -2.5 |
| cadherin, EGF LAG seven-pass G-type receptor 2 (flamingo homolog, Drosophila) | Celsr2 | -2.5 |
| itchy, E3 ubiquitin protein ligase | Itch | -2.4 |
| nudix (nucleoside diphosphate linked moiety X)-type motif 18 | Nudt18 | -2.4 |
| RAB21, member RAS oncogene family | Rab21 | -2.3 |
| epsin 1 | Epn1 | -2.3 |
| kinesin family member 21A | Kif21a | -2.2 |
| latrophilin 1 | Lphn1 | -2.2 |
| actin related protein 2/3 complex, subunit 4 | Arpc4 | -2.1 |
| RIKEN cDNA 2010007H12 gene | 2010007H12Rik | -1.8 |
| thiosulfate sulfurtransferase, mitochondrial | Tst | -1.7 |
| alanyl (membrane) aminopeptidase | Anpep | -1.6 |
| acyl-CoA thioesterase 7 | Acot7 | -1.4 |

Most 25 up and 24 down regulated genes found in the kidney tissue to be regulated by ischemic pre-conditioning (IPC+IRI *vs* IRI). Gene expression fold changes are represented by IPC+IRI group gene expression values in relation to IRI values.
