# Supplementary material for: Transcriptome Analysis of Renal Ischemia/Reperfusion Injury and Its Modulation by Ischemic Pre-Conditioning or Hemin Treatment
Source: PLoS One. 2012 Nov 14;7(11):e49569. doi: 10.1371/journal.pone.0049569 (PMC3498198; doi:10.1371/journal.pone.0049569)
Supplement: Table S3 — Gene profile comparison between IPC and control groups. (DOC) [file pone.0049569.s003.doc]

**Table S3**. Gene profile comparison between IPC and control groups.

| | **Name** | **Symbol** | **Fold change** | | --- | --- | --- | | chemokine (C-X-C motif) ligand 1 | Cxcl1 | 94.0 | | activating transcription factor 3 | Atf3 | 69.9 | | v-maf musculoaponeurotic fibrosarcoma oncogene family, protein F (avian) | Maff | 68.6 | | fos-like antigen 1 | Fosl1 | 47.1 | | suppressor of cytokine signaling 3 | Socs3 | 45.3 | | tumor necrosis factor receptor superfamily, member 12a | Tnfrsf12a | 42.0 | | cyclin-dependent kinase inhibitor 1A (P21) | Cdkn1a | 33.3 | | growth arrest and DNA-damage-inducible 45 beta | Gadd45b | 32.0 | | sphingosine kinase 1 | Sphk1 | 31.7 | | S100 calcium binding protein A8 (calgranulin A) | S100a8 | 31.5 | | SRY-box containing gene 9 | Sox9 | 30.5 | | metallothionein 1 | Mt1 | 25.4 | | cholesterol 25-hydroxylase | Ch25h | 21.7 | | Eph receptor A2 | Epha2 | 20.9 | | ChaC, cation transport regulator-like 1 (E. coli) | Chac1 | 19.9 | | similar to gag protein | LOC100047599 | 19.8 | | absent in melanoma 1-like | Aim1l | 18.4 | | 28S ribosomal RNA | Rn28s1 | 18.4 | | interferon-related developmental regulator 1 | Ifrd1 | 17.1 | | RNA binding motif protein 39 | Rbm39 | 16.8 | | methyl-CpG binding domain protein 1 | Mbd1 | 16.5 | | plasminogen activator, urokinase receptor | Plaur | 16.5 | | RIKEN cDNA 1700024P16 gene | 1700024P16Rik | 16.4 | | mitogen-activated protein kinase kinase kinase 6 | Map3k6 | 16.4 | | polo-like kinase 3 (Drosophila) | Plk3 | 16.0 | | solute carrier family 4, sodium bicarbonate cotransporter, member 7 | Slc4a7 | -19.8 | | SRY-box containing gene 18 | Sox18 | -12.7 | | O-linked N-acetylglucosamine (GlcNAc) transferase (UDP-N-acetylglucosamine:polypeptide-N-acetylglucosaminyl transferase) | Ogt | -12.4 | | avian musculoaponeurotic fibrosarcoma (v-maf) AS42 oncogene homolog | Maf | -6.8 | | SUMO/sentrin specific peptidase 8 | Senp8 | -6.8 | | K+ voltage-gated channel, subfamily S, 2 | Kcns2 | -6.2 | | superoxide dismutase 3, extracellular | Sod3 | -6.1 | | H19 fetal liver mRNA | H19 | -6.0 | | olfactory receptor 225 | Olfr225 | -6.0 | | B cell leukemia/lymphoma 11B | Bcl11b | -6.0 | | olfactory receptor 691 | Olfr691 | -5.8 | | RIKEN cDNA E230016K23 gene | E230016K23Rik | -5.8 | | protein phosphatase 2, regulatory subunit B (B56), epsilon isoform | Ppp2r5e | -5.5 | | karyopherin (importin) alpha 4 | Kpna4 | -5.3 | | PDZ domain containing 7 | Pdzd7 | -5.2 | | FYVE, RhoGEF and PH domain containing 4 | Fgd4 | -5.1 | | RIKEN cDNA 4933406B17 gene | 4933406B17Rik | -5.0 | | olfactory receptor 1280 | Olfr1280 | -4.9 | | olfactory receptor 161 | Olfr161 | -4.9 | | RIKEN cDNA 5730522E02 gene | 5730522E02Rik | -4.9 | | von Willebrand factor A domain containing 5B2 | Vwa5b2 | -4.8 | | IKAROS family zinc finger 4 | Ikzf4 | -4.7 | | solute carrier family 9 (sodium/hydrogen exchanger), member 4 | Slc9a4 | -4.7 | | ribosomal RNA processing 1 homolog B (S. cerevisiae) | Rrp1b | -4.7 | | HRAS-like suppressor family, member 5 | Hrasls5 | -4.6 | |
| --- | --- | --- | --- | --- | --- | --- | --- | --- | --- | --- | --- | --- | --- | --- | --- | --- | --- | --- | --- | --- | --- | --- | --- | --- | --- | --- | --- | --- | --- | --- | --- | --- | --- | --- | --- | --- | --- | --- | --- | --- | --- | --- | --- | --- | --- | --- | --- | --- | --- | --- | --- | --- | --- | --- | --- | --- | --- | --- | --- | --- | --- | --- | --- | --- | --- | --- | --- | --- | --- | --- | --- | --- | --- | --- | --- | --- | --- | --- | --- | --- | --- | --- | --- | --- | --- | --- | --- | --- | --- | --- | --- | --- | --- | --- | --- | --- | --- | --- | --- | --- | --- | --- | --- | --- | --- | --- | --- | --- | --- | --- | --- | --- | --- | --- | --- | --- | --- | --- | --- | --- | --- | --- | --- | --- | --- | --- | --- | --- | --- | --- | --- | --- | --- | --- | --- | --- | --- | --- | --- | --- | --- | --- | --- | --- | --- | --- | --- | --- | --- | --- | --- | --- | --- |

Most 25 up and 25 down regulated genes found in the kidney tissue after ischemia pre-conditioning and ischemia/reperfusion injury (IPC+IRI *vs* Control). Gene expression fold changes are represented by IPC+IRI group gene expression values in relation to Control values.
