# Supplementary material for: Transcriptome Analysis of Renal Ischemia/Reperfusion Injury and Its Modulation by Ischemic Pre-Conditioning or Hemin Treatment
Source: PLoS One. 2012 Nov 14;7(11):e49569. doi: 10.1371/journal.pone.0049569 (PMC3498198; doi:10.1371/journal.pone.0049569)
Supplement: Table S4 — Gene profile comparison between Hemin+IRI and IRI groups. (DOC) [file pone.0049569.s004.doc]

**Table S4**. Gene profile comparison between Hemin+IRI and IRI groups.

| | **Name** | **Symbol** | **Fold change** | | --- | --- | --- | | tripartite motif-containing 55 | Trim55 | 23.2 | | 28S ribosomal RNA | Rn28s1 | 19.8 | | DEAD (Asp-Glu-Ala-Asp) box polypeptide 25 | Ddx25 | 15.4 | | solute carrier family 7 (cationic amino acid transporter, y+ system), member 12 | Slc7a12 | 15.1 | | serine/arginine repetitive matrix 4 | Srrm4 | 11.5 | | neuregulin 1 | Nrg1 | 11.3 | | synaptic nuclear envelope 2 | Syne2 | 11.0 | | ELOVL family member 7, elongation of long chain fatty acids (yeast) | Elovl7 | 10.7 | | prolactin receptor | Prlr | 10.0 | | synaptotagmin X | Syt10 | 9.5 | | 2-hydroxyacyl-CoA lyase 1 | Hacl1 | 9.3 | | Riken cDNA A630031M23 gene | A630031M23Rik | 9.2 | | golgi autoantigen, golgin subfamily b, macrogolgin 1 | Golgb1 | 9.1 | | fibrinogen gamma chain | Fgg | 9.1 | | sema domain, transmembrane domain (TM), and cytoplasmic domain, (semaphorin) 6C | Sema6c | 8.9 | | plectin | Plec | 8.8 | | H1 histone family, member X | H1fx | 8.8 | | nuclear receptor subfamily 2, group C, member 2 | Nr2c2 | 8.7 | | leucyl-tRNA synthetase | Lars | 8.7 | | fibroblast growth factor 18 | Fgf18 | 8.3 | | family with sequence similarity 168, member A | Fam168a | 8.3 | | DEAD (Asp-Glu-Ala-Asp) box polypeptide 46 | Ddx46 | 8.1 | | RIKEN cDNA 4931406H21 gene | 4931406H21Rik | 8.1 | | CUB domain containing protein 1 | Cdcp1 | 8.0 | | membrane magnesium transporter 2 | Mmgt2 | 7.9 | | solute carrier family 4, sodium bicarbonate cotransporter, member 7 | Slc4a7 | -17.6 | | spermatogenesis associated glutamate (E)-rich protein 5, pseudogene 1 | Speer5-ps1 | -9.6 | | chemokine (C-C motif) ligand 5 | Ccl5 | -7.5 | | kinesin family member 20B | Kif20b | -6.6 | | WD repeat domain 70 | Wdr70 | -5.9 | | mitogen-activated protein kinase kinase 6 | Map2k6 | -5.7 | | superoxide dismutase 3, extracellular | Sod3 | -5.7 | | formin-like 1 | Fmnl1 | -5.6 | | netrin 1 | Ntn1 | -5.5 | | 3-hydroxy-3-methylglutaryl-Coenzyme A synthase 1 | Hmgcs1 | -5.4 | | predicted gene 9758 | Gm9758 | -5.3 | | solute carrier family 8 (sodium/calcium exchanger), member 1 | Slc8a1 | -5.2 | | karyopherin (importin) alpha 4 | Kpna4 | -5.2 | | cryptochrome 1 (photolyase-like) | Cry1 | -5.0 | | ankyrin repeat and sterile alpha motif domain containing 1B | Anks1b | -5.0 | | transmembrane emp24 protein transport domain containing 7 | Tmed7 | -5.0 | | SEC23A (S. cerevisiae) | Sec23a | -4.9 | | profilin 1 | Pfn1 | -4.8 | | PRKC, apoptosis, WT1, regulator | Pawr | -4.7 | | RAP2B, member of RAS oncogene family | Rap2b | -4.6 | | solute carrier family 33 (acetyl-CoA transporter), member 1 | Slc33a1 | -4.6 | | nuclear receptor subfamily 3, group C, member 1 | Nr3c1 | -4.5 | | SH3-domain GRB2-like B1 (endophilin) | Sh3glb1 | -4.2 | | pigeon homolog (Drosophila) | Pion | -3.9 | | heterogeneous nuclear ribonucleoprotein L | Hnrnpl | -3.9 | |  |
| --- | --- | --- | --- | --- | --- | --- | --- | --- | --- | --- | --- | --- | --- | --- | --- | --- | --- | --- | --- | --- | --- | --- | --- | --- | --- | --- | --- | --- | --- | --- | --- | --- | --- | --- | --- | --- | --- | --- | --- | --- | --- | --- | --- | --- | --- | --- | --- | --- | --- | --- | --- | --- | --- | --- | --- | --- | --- | --- | --- | --- | --- | --- | --- | --- | --- | --- | --- | --- | --- | --- | --- | --- | --- | --- | --- | --- | --- | --- | --- | --- | --- | --- | --- | --- | --- | --- | --- | --- | --- | --- | --- | --- | --- | --- | --- | --- | --- | --- | --- | --- | --- | --- | --- | --- | --- | --- | --- | --- | --- | --- | --- | --- | --- | --- | --- | --- | --- | --- | --- | --- | --- | --- | --- | --- | --- | --- | --- | --- | --- | --- | --- | --- | --- | --- | --- | --- | --- | --- | --- | --- | --- | --- | --- | --- | --- | --- | --- | --- | --- | --- | --- | --- | --- | --- |

Most 25 up and 25 down regulated genes found in the kidney tissue after ischemia-reperfusion injury in mice previously treated with Hemin (IRI+Hemin *vs* IRI). Gene expression fold changes are represented by IRI+Hemin group gene expression values in relation to IRI values.
