# Supplementary material for: Transcriptome Analysis of Renal Ischemia/Reperfusion Injury and Its Modulation by Ischemic Pre-Conditioning or Hemin Treatment
Source: PLoS One. 2012 Nov 14;7(11):e49569. doi: 10.1371/journal.pone.0049569 (PMC3498198; doi:10.1371/journal.pone.0049569)
Supplement: Table S5 — Gene profile comparison between Hemin and Control groups. (DOC) [file pone.0049569.s005.doc]

**Table S5**. Gene profile comparison between Hemin and Control groups.

| | **Name** | **Symbol** | **Fold change** | | --- | --- | --- | | 28S ribosomal RNA | Rn28s1 | 18.8 | | 18S ribosomal RNA | Rn18s | 14.5 | | WD repeat domain 16 | Wdr16 | 7.7 | | histone cluster 3, H2ba | Hist3h2ba | 6.4 | | ferritin, heavy polypeptide-like 17 | Fthl17 | 6.4 | | uroplakin 3B | Upk3b | 6.0 | | lamin B1 | Lmnb1 | 6.0 | | neuron navigator 3 | Nav3 | 5.8 | | calmodulin-like 3 | Calml3 | 5.7 | | homeobox D4 | Hoxd4 | 5.7 | | tudor domain containing 12 | Tdrd12 | 5.7 | | synaptotagmin X | Syt10 | 5.6 | | RNA binding motif protein 39 | Rbm39 | 5.2 | | sperm associated antigen 5 | Spag5 | 5.2 | | preproenkephalin | Penk | 5.1 | | nuclear factor of activated T cells 5 | Nfat5 | 5.1 | | cholinergic receptor, muscarinic 1, CNS | Chrm1 | 4.9 | | RWD domain containing 2A | Rwdd2a | 4.9 | | arginase type II | Arg2 | 4.9 | | protein tyrosine phosphatase, receptor type, B | Ptprb | 4.8 | | zinc finger, MYND domain containing 19 | Zmynd19 | 4.8 | | apolipoprotein A-IV | Apoa4 | 4.8 | | RIKEN cDNA 6430598A04 gene | 6430598A04Rik | 4.8 | | zinc finger, FYVE domain containing 9 | Zfyve9 | 4.8 | | mucin 20 | Muc20 | 4.7 | |
| --- | --- | --- | --- | --- | --- | --- | --- | --- | --- | --- | --- | --- | --- | --- | --- | --- | --- | --- | --- | --- | --- | --- | --- | --- | --- | --- | --- | --- | --- | --- | --- | --- | --- | --- | --- | --- | --- | --- | --- | --- | --- | --- | --- | --- | --- | --- | --- | --- | --- | --- | --- | --- | --- | --- | --- | --- | --- | --- | --- | --- | --- | --- | --- | --- | --- | --- | --- | --- | --- | --- | --- | --- | --- | --- | --- | --- | --- | --- |

Most 25 up regulated genes found in the kidney tissue after treatment with Hemin (Hemin *vs* Control). Gene expression fold changes are represented by Hemin group gene expression values in relation to Control values.
