# Supplementary material for: Transcriptome Analysis of Renal Ischemia/Reperfusion Injury and Its Modulation by Ischemic Pre-Conditioning or Hemin Treatment
Source: PLoS One. 2012 Nov 14;7(11):e49569. doi: 10.1371/journal.pone.0049569 (PMC3498198; doi:10.1371/journal.pone.0049569)
Supplement: Table S6 — Up regulated genes in IRI group (vs control), according to GO and KEGG categories. (DOC) [file pone.0049569.s006.doc]

**Table S6.** Up regulated genes in IRI group (vs control), according to GO and KEGG categories.

| **CATEGORIES** | **Differentially expressed genes** |
| --- | --- |
| **Positive regulation of cell proliferation** | Camk2d, Adm, Atf3, Bcl2l1, Cdkn1a, Csf1, S1pr1, Edn1, Egr1, Fgfbp1, Ctgf, Fosl2, Hbegf, Hipk1, Hmox1, Cxcl10, Cyr61, Ihh, Itgav, Itgb3, Jun, Myc, Ngf, Osmr, Pdgfa, Rela, Sox9, Sphk1, Nrg1, Tsc22d1, Tgif1, Tnc, Acer2, Fgf21, Clcf1, Rasip1, Il34 |
| **Apoptosis** | Birc3, Rhob, Bcl2l1, Cryaa, Epha2, F3, Fas, Fem1b, Gata6, Krt18, Krt8, Lcn2, Bbc3, Mcl1, Mef2d, Myc, Ppp1r15a, Gadd45b, Ngf, Klf11, Serpina3g, Csrnp1, Phlda1, Tnfaip3, Trib3, Tnfrsf12a, Bag3, Fgd3, Sfn, vRybp, Litaf, Nek6, Trp53inp1, Chac1, Irak3, Clca2 |
| **Cell differentiation** | Kdm3a, Rhob, Bcl3, Cebpb, Col13a1, Elf3, Ctgf, Gna13, Ifrd1, Cyr61, Ihh, Itgav, Junb, Klf4, Lgals3, Mcl1, Mdfi, Mef2d, Sik1, Gadd45b, Nfkb2, Nrp1, Odf2, Rora, Sema4c, Sema6b, Sema7a, Slc7a5, Sox9, Tnfrsf12a, Pdlim7 |
| **Angiogenesis** | Adamts1, Rhob, Klf5, Btg1, Anxa2, S1pr1, Edn1, Epha2, F3, Ctgf, Gata6, Gna13, Hbegf, Hmox1, Cxcl10, Cyr61, Ihh, Itgav, Jun, Nos3, Nrp1, Pdgfa, Serpine1, Ccl2, Sphk1, Thbs1, Tnfrsf12a, Rtn4 |
| **Cell adhesion** | Abl2, Rhob, Azgp1, Cd44, Col13a1, Cxadr, Ctgf, Icam1, Cyr61, Itga5, Itgav, Itgb3, Lamb3, Lamc2, Cd93, Nrp1, Tgfbi, Thbs1, Tnc, Vcam1, Emilin2, Amica1, Tnfrsf12a, Pkp3 |
| **Inflammatory response** | Adam8, Cd14, Cd44, Elf3, Cxcl1, Hmox1, Cxcl10, Il1b, Rela, Ccl2, Sema7a, Sphk1, Thbs1, Tnfrsf1b, Map2k3, Nfkbiz |
| **Response to stress** | Adm, Cirbp, Hspb1, Krt8, Lcn2, Ppp1r15a, Nr4a2, Rbm3, Uchl1, Map4k4, Trp53inp1, Fam129a, Hilpda |
| **small GTPase mediated signal transduction** | Rhob, Gem, Hmox1, Ngf, Rasd1, Rnd1, Arl4c, Rhou, Rap2b, Rnd3, Rab30, Rhoj |
| **I-kappaB kinase/NF-kappaB cascade** | Bcl3, Hmox1, Il1b, Nfkbia, Slc20a1, Tnfaip3, Tnip2, Litaf, Nek6, Ndfip2, Zc3hav1 |
| **MAPK signaling pathway** | Rps6ka3, Rasa2, Atf4, Cd14, Crk, Gadd45a, Ddit3, Dusp2, Fas, Fos, Nr4a1, Hspb1, Il1b, Il1r1, Jun, Myc, Gadd45b, Nfkb2, Ngf, Dusp8, Pdgfa, Dusp1, Rela, Map2k3, Map3k2, Map3k8, Map4k4, Dusp4, Map3k6, Dusp14, Fgf21, Dusp10, Flnc |
| **Pathways in cancer** | Birc3, Bcl2l1, Cdkn1a, Crk, Fas, Fos, Itgav, Jun, Lamb3, Lamc2, Myc, Nfkb2, Nfkbia, Pdgfa, Rela, Stat3, Wnt10a, Pik3r5, Fgf21 |
| **Cytokine-cytokine receptor interaction** | Csf1, Faz, Cxcl1, Cxcl10, Il1b, Il1r1, Il4ra, Inhba, Inhbb, Osmr, Pdgfa, Ccl9, Il20rb, Tnfrsf1b, Tnfrsf12a, Clcf1 |
| **Focal adhesion** | Birc3, Crk, Itga5, Itgav, Itgb3, Jun, Lamb3, Lamc2, Pdgfa, Rock2, Thbs1, Tnc, Pik3r5, Flnc |
| **Neurotrophin signaling pathway** | Camk2d, Rps6ka3, Atf4, Crk, Jun, Nfkbia, Ngf, Rela, Pik3r5, Calml3, Irak3 |
| **Regulation of actin cytoskeleton** | Cd14, Crk, Gna13, Itga5, Itgav, Itgb3, Pdgfa, Rock2, Fgd3, Pik3r5, Fgf21 |
| [**ErbB signaling pathway**](http://www.genome.jp/kegg-bin/mark_pathway_www?@mmu04012/default%3Dwhite/reference%3D%23FFFF33/11352%09red,black/12575%09red,black/12928%09red,black/13685%09red,black/15200%09red,black/16476%09red,black/17869%09red,black/320207%09red,black/) | Abl2, Cdkn1a, Crk, Eif4ebp1, Hbegf, Jun, Myc, Pik3r5, Camk2d, Nrg1 |
| **Jak-STAT signaling pathway** | Bcl2l1, Socs3, Il4ra, Myc, Osmr, Pim1, Stat3, Il20rb, Pik3r5, Clcf1 |
| **Toll-like receptor signaling pathway** | Cd14, Fos, Cxcl10, Il1b, Jun, Nfkbia, Rela, Map2k3, Map3k8, Pik3r5 |
| **Chemokine signaling pathway** | Crk, Cxcl1, Cxcl10, Nfkbia, Rela, Rock2, Ccl2, Ccl9, Stat3, Pik3r5 |
| **Hematopoietic cell lineage** | Cd14, Cd44, Csf1, Il1b, Il1r1, Il4ra, Itga5, Itgb3 |
| **ECM-receptor interaction** | Cd44, Itga5, Itgav, Itgb3, Lamb3, Lamc2, Thbs1, Tnc |
| **p53 signaling pathway** | Cdkn1a, Gadd45a, Fas, Bbc3, Gadd45b, Serpine1, Thbs1, Sfn |
| **Axon Guidance** | Epha2, Nrp1, Plxna2, Rock2, Sema4c, Sema6b, Sema7a, Rnd1 |
| **NOD-like receptor signaling pathway** | Birc3, Cxcl1, Il1b, Nfkbia, Rela, Tnfaip3 |
| [**Adipocytokine signaling pathway**](http://www.genome.jp/kegg-bin/mark_pathway_www?@mmu04920/default%3Dwhite/reference%3D%23FFFF33/12702%09red,black/18035%09red,black/19697%09red,black/20525%09red,black/20848%09red,black/21938%09red,black/50790%09red,black/) | Socs3, Nfkbia, Rela, Stat3, Tnfrsf1b, Acsl4 |
| **Complement and coagulation cascades** | F3, Masp1, Serpine1, Plat, Plaur, Thbd |
| [**Sphingolipid metabolism**](http://www.genome.jp/kegg-bin/mark_pathway_www?@mmu00600/default%3Dwhite/reference%3D%23FFFF33/20698%09red,black/22234%09red,black/230379%09red,black/56386%09red,black/74442%09red,black/) | Sphk1, Ugcg, Acer2, B4galt6, Sgms2 |
| **Circadian rhythm - mammal** | Csnk1d, Cry1, Rora, Bhlhe40 |
| **Mineral Absorption** | Hmox1, Mt1, Mt2, Trpv6 |

Differentially up-regulated genes after ischemia-reperfusion injury (IRI x Control) classified in the most relevant GO and KEGG categories.
