# Supplementary material for: Transcriptome Analysis of Renal Ischemia/Reperfusion Injury and Its Modulation by Ischemic Pre-Conditioning or Hemin Treatment
Source: PLoS One. 2012 Nov 14;7(11):e49569. doi: 10.1371/journal.pone.0049569 (PMC3498198; doi:10.1371/journal.pone.0049569)
Supplement: Table S7 — Down regulated genes in IRI group (vs control), according to GO and KEGG categories. (DOC) [file pone.0049569.s007.doc]

**Table S7.** Down regulated genes in IRI group (vs control), according to GO and KEGG categories.

| **CATEGORIES** | **Differentially expressed genes** |
| --- | --- |
| **Olfactory Transduction** | Olfr60, Olfr149, Olfr1359, Olfr247, Olfr1232, Olfr1317, Olfr1215, Olfr1170, Olfr297, Olfr418-ps1, Olfr441, Olfr478, Olfr821, Olfr1230, Olfr620, Olfr103, Olfr1206, Olfr791, Olfr338, Olfr1226, Olfr139, Olfr1122, Olfr979, Olfr1371, Olfr1507 |
| **Ion Transport** | Slc9a4, Atp7b, Grin2b, Kcns2, Slc13a2, Slc34a2, Kcnk12, Nipal4, Trpc3, P2rx3, Slc5a12, Ano8, Gria3, Slc4a4, Kcnc4 |
| **nervous system development** | Prrxl1, Cit, Efna5, Fut9, Ascl1, Sema5b, Sim1, Slit2, Insc |
| **Neuroactive ligand-receptor interaction** | Agtr1b, Avpr2, Bdkrb1, Grin2b, Htr2b, Prlr, Tbxa2r, P2rx3, Aplnr, Gria3, 2210010C04Rik |
| **MAPK signaling pathway** | Cdc25b, Crkl, Fgf11, Prkcb, Rasgrp2, Pla2g3, Map3k12, Taok2, Nfatc4, Dusp9 |
| **Pancreatic secretion** | Ctrl, Prkcb, Pla2g3, Slc4a4, Ctrb1, 2210010C04Rik |
| **Arachidonic acid metabolism**  **Glutamatergic synapse** | Cyp2b13, Tbxas1, Pla2g3, Plb1, Ptges2  Gls, Grin2b, Prkcb, Pla2g3, Gria3 |
| **cell projection organization** | Dync2h1, Capzb, Crocc, Whrn, Vangl2 |
| **Protein digestion and absorption** | Ctrl, Col2a1, Ctrb1, 2210010C04Rik |
| **activation of protein kinase activity** | Bdkrb1, Htr2b, Prkcz, Prlr, Taok2 |
| **ErbB signaling pathway** | Crkl, Prkcb, Pak6, Shc4 |
| **Renin-angiotensin system** | Agtr1b, Cma1 |
| **Glycosphingolipid biosynthesis - globo series** | Fut9, B3galnt1 |
| **Proximal tubule bicarbonate reclamation** | Gls, Slc4a4 |
| **D-Glutamine and D-glutamate metabolism** | Gls |

Differentially down-regulated genes after ischemia-reperfusion injury (IRI x Control) classified in the most relevant GO and KEGG categories.
