# Supplementary material for: Transcriptome Analysis of Renal Ischemia/Reperfusion Injury and Its Modulation by Ischemic Pre-Conditioning or Hemin Treatment
Source: PLoS One. 2012 Nov 14;7(11):e49569. doi: 10.1371/journal.pone.0049569 (PMC3498198; doi:10.1371/journal.pone.0049569)
Supplement: Table S8 — Up regulated genes in IPC group (vs IRI), according to GO and KEGG categories. (DOC) [file pone.0049569.s008.doc]

**Table S8.** Up regulated genes in IPC group (vs IRI), according to GO and KEGG categories.

| **CATEGORIES** | **DIFFERENTIALLY EXPRESSED GENES** |
| --- | --- |
| RNA transport and surveillance pathway | Tpr, Eif5b, Upf2, Acin1, Eif3c, Upf3b, Smg6, Upf3b |
| Protein processing in endoplasmic reticulum | Bax, Ckap4, Hsp90b1, Fbxo2, Uggt1 |
| Systemic lupus erythematosus | Hist1h4d, Hist1h4i, Hist3h2a, Hist2h2be, Hist3h2ba |
| Steroid hormone biosynthesis | Hsd17b7, Hsd17b12, Hsd3b4, Srd5a1 |
| Transcription | Ddx1, Chd9, Nr2f2, Eya3, Hoxa7, Nfix, Med1, Ppargc1a, Tcf3, ef, Thra, Nr2c1, Zfp148, Eif2c1, Taf7, Taf1, Zfp292, Snapc5, Banp, Nfat5, Ccar1, Zfp397. Mier1, Mterfd3, Zkscan1 |
| nervous system development | Ccdc88a, Bax, Fut9, Nedd4, Sema6c, Apob, Ghrl |
| RNA splicing | Srpk2, Fus, Zranb2, Ppih, Ccar1, Upf3b, Cdc40 |
| spermatogenesis | Adcyap1r1, Bax, Apob, Ddx25, Styx, Hook1 |
| epithelial cell proliferation | Nr2f2, Bax, Fabp7, Cdkn1b, Med1, Ghrl |
| cellular membrane organization | Gbf1, Ccdc88a, Bax, Hspa4, Acrn1 |
| microtubule cytoskeleton organization | Ccdc88a, Cdk5rap2, Hook3, Clasp1, Hook1 |
| DNA replication | Ccdc88a, Nfix, Pola1, Med1, Orc3 |
| protein folding | Hsp90b1, Ppig, Uggt1, Ppig, Pfdn1 |

Differentially up-regulated genes modulated by ischemic preconditioning (IPC+IRI x IRI) classified in the most relevant GO and KEGG categories.
