# Supplementary material for: Transcriptome Analysis of Renal Ischemia/Reperfusion Injury and Its Modulation by Ischemic Pre-Conditioning or Hemin Treatment
Source: PLoS One. 2012 Nov 14;7(11):e49569. doi: 10.1371/journal.pone.0049569 (PMC3498198; doi:10.1371/journal.pone.0049569)
Supplement: Table S9 — Down regulated genes in IPC group (vs IRI), according to GO and KEGG categories. (DOC) [file pone.0049569.s009.doc]

**Table S9.** Down regulated genes in IPC group (vs IRI), according to GO and KEGG categories.

| **CATEGORIES** | **DIFFERENTIALLY EXPRESSED GENES** |
| --- | --- |
| Endocytosis | Epn1, Itch |
| Cell cycle | Myc, Ep300 |
| Aminoacid metabolism | Cndp1 |
| Regulation of actin cytoskeleton | Pfn1, Arpc4 |
| Renin-angiotensin system | Anpep |
| Sulfur relay system | Tst |
| Biosynthesis of unsaturated fatty acids | Acot7 |
| Wnt, TGF-β and JAK-STAT signaling pathway | Myc, Ep300, Celsr2 |
| apoptosis | Itch, Myc, Ep300 |
| response to hypoxia | Ep300, Sod3 |
| response to metal ion | Ep300, Sod3 |

Differentially down-regulated genes modulated by ischemic preconditioning (IPC+IRI x IRI) classified in the most relevant GO and KEGG categories.
