# Supplementary material for: Transcriptome Analysis of Renal Ischemia/Reperfusion Injury and Its Modulation by Ischemic Pre-Conditioning or Hemin Treatment
Source: PLoS One. 2012 Nov 14;7(11):e49569. doi: 10.1371/journal.pone.0049569 (PMC3498198; doi:10.1371/journal.pone.0049569)
Supplement: Table S10 — Up regulated genes in IPC group (vs control), according to GO and KEGG categories. (DOC) [file pone.0049569.s010.doc]

**Table S10.** Up regulated genes in IPC group (vs control), according to GO and KEGG categories.

| **CATEGORIES** | **Differentially expressed genes** |
| --- | --- |
| **Apoptosis** | Topors, Jmjd6, Birc3, Eif2ak3, Epha2, F3, Fas, Fem1b, Krt18, Krt8, Lcn2, Mcl1, Myc, Ppp1r15a, Gadd45b, Nfkb1, Sgk1, Serpina3g, Csrnp1, Rnf144b, Tnfaip3, Trib3, Zc3h12a, Tnfrsf12a, Bag3, Fgd3, Sfn, Acin1, Rybp, Litaf, Nek6, Trp53inp1, Nisch, Rbm25, Chac1, Ddit4, Akap13 |
| **Positive regulation of cell proliferation** | Camk2d, Atf3, Cdkn1a, Csf1, Egr1, Fgfbp1, Fosl2, Hbegf, Hmox1, Cxcl10, Cyr61, Ihh, Itgav, Cd47, Jun, AY036118, Myc, Osmr, Pdgfa, Rela, Sox4, Sox9, Sphk1, Nrg1, Acer2, Il34 |
| **Angiogenesis** | Adamts1, Klf5, C3, Anxa2, Epha2, F3, Fzd5, Hbegf, Hmox1, Cxcl10, Cyr61, Ihh, Itgav, Jun, Nrp1, Pdgfa, Serpine1, Ptprb, Ccl2, Sphk1, Zc3h12a, Wasf2, Tnfrsf12a, Foxo6, Rtn4 |
| **Negative regulation of apoptosis** | Bcl3, Btg2, Cd44, Cdkn1a, Fas, Hspb1, Hspa1b, Cyr61, Ihh, Itgav, Jun, Krt18, Anxa1, Mcl1, Serpine1, Plaur, Sgk1, Sox4, Sox9, Sphk1, Timp1, Tnfaip3, Asns, Bag3 |
| **inflammatory response** | Adam8, C3, Cd14, Cd44, Cxcl1, Hmox1, Cxcl10, Il1b, Rela, Ccl2, Sema7a, Sphk1, Tnfrsf1b, Tlr2,Map2k3, Nfkbiz |
| **positive regulation of intracellular protein kinase cascade** | F3, Hbegf, Hmox1, Il1b, Lmnb1, Pdgfa, Sema4c, Slc20a1, Ubd, Litaf, Nek6, Mier1, Zc3hav1, Akap12 |
| **I-kappaB kinase/NF-kappaB cascade** | Bcl3, Hmox1, Il1b, Nfkbia, Slc20a1, Tnfaip3, Otud7b, Tlr2, Ubd, Litaf, Nek6, Mier1, Zc3hav1 |
| **response to stress** | Cirbp, Eif2ak3, Hspb1, Hspa1b, Krt8, Lcn2, Ppp1r15a, Nr4a2, Hspa1a, Sgk1, Trp53inp1, Hilpda |
| **regulation of cell differentiation** | Socs3, Csf1, Gnas, Cyr61, Ihh, Itgav, Klf4, Nfkbia, Sox9, Nrg1, Rod1, Apob |
| **blood vessel development** | Jmjd6, Socs3, Epha2, Fzd5, Cyr61, Itgav, Junb, Sphk1, Chd7 |
| **platelet-derived growth factor receptor signaling pathway** | F3, Pdgfa, Plat, Csrnp1, Zfand5, Arid5b, Tiparp |
| **tumor necrosis factor-mediated signaling pathway** | Fas, Krt18, Krt8, Sphk1, Tnfrsf1b |
| **cellular response to metal ion** | Fas, Fos, Fosb, Jun, Junb |
| **MAPK signaling pathway** | Atf4, Cd14, Crk, Gadd45a ,Ddit3, Dusp2, Fas, Fos, Nr4a1, Hspb1, Hspa1b, Il1b, Jun, Myc, Gadd45b, Nfkb1, Dusp8, Pdgfa, Hspa1a, Rela, Relb, Dusp7, Map2k3, Map3k8, Dusp4, Map3k6, Dusp14, Dusp10, Flnc |
| **Pathways in Cancer** | Birc3, Cdkn1a, Crk, Fas, Fos, Fzd5, Itga6, Itgav, Jun, Myc, Nfkb1, Nfkbia, Pdgfa, Rela, Ralgds, Stat3, Wnt10a, Pik3r5 |
| **Cytokine-cytokine receptor interaction** | Csf1, Faz, Cxcl1, Cxcl10, Il12rb1, Il1b, Il4ra, Inhbb, Osmr, Pdgfa, Ccl17, Il20rb, Tnfrsf1b, Tnfrsf12a |
| **Toll-like receptor signaling pathway** | Cd14, Cd86, Fos, Cxcl10, Il1b, Jun, Nfkb1, Nfkbia, Rela, Tlr2, Map2k3, Map3k8, Pik3r5 |
| **Wnt signaling pathway** | Camk2d, Fosl1, Fzd5, Jun, Mmp7, Myc, Rock2, Frat2, Wnt10a, Csnk1e, Nfat5, Ppp2r1b |
| **Focal adhesion** | Actb, Birc3, Cav3, Crk, Itga6, Itgav, Jun, Pdgfa, Rock2, Pik3r5, Flnc |
| **Chemokine signaling pathway** | Crk, Cxcl1, Cxcl10, Nfkb1, Nfkbia, Rela, Rock2, Ccl17, Ccl2, Stat3, Pik3r5 |
| **Spliceosome** | Sf3b3, U2af1, Hspa1b, Hspa1a, Srsf5, Hnrnpa3,Acin1, Rbm25, Snrpd3, Ddx23 |
| **Phagosome** | Actb, Atp6v0a1, C3, Cd14, Ctss, Itgav, Tubb2a, 5430435G22Rik, Tlr2, Tubb6 |
| **ErbB signaling pathway** | Camk2d, Abl2, Cdkn1a, Crk, Hbegf, Jun, Myc, Nrg1, Pik3r5 |
| **Neurotrophin signaling pathway** | Camk2d, Atf4, Crk, Jun, Nfkb1, Nfkbia, Rela, Pik3r5, Calml3 |
| **NOD-like receptor signaling pathway** | Birc3, Cxcl1, Il1b, Nfkb1, Nfkbia, Rela, Tnfaip3, Erbb2ip |
| **Rheumatoid arthritis** | Atp6v0a1, Cd86, Csf1, Fos, Icam1, Il1b, Jun, Tlr2 |
| **Axon guidance** | Epha2, Nrp1, Rock2, Sema4c, Sema7a, Rnd1,Ablim1, Nfat5 |
| **p53 signaling pathway** | Cdkn1a, Gadd45a, Sesn1, Fas, Gadd45b, Serpine1, Sfn |
| **Adipocytokine signaling pathway** | Socs3, Nfkb1, Nfkbia, Rela, Stat3, Tnfrsf1b, Acsl4 |
| **Complement and coagulation cascades** | C3, F3, Serpine1, Plat, Plaur, Fgg |
| **Hematopoietic cell lineage** | Cd14, Cd44, Csf1, Il1b, Il4ra, Itga6 |
| **Cytosolic DNA-sensing pathway** | Cxcl10, Il1b, Nfkb1, Nfkbia, Rela, Polr3f |
| **Circadian rhythm - mammal** | Csnk1d, Per1, Csnk1e |

Differentially up-regulated genes after ischemic preconditioning (IPC+IRI x Control) classified in the most relevant GO and KEGG categories.
