# Supplementary material for: Transcriptome Analysis of Renal Ischemia/Reperfusion Injury and Its Modulation by Ischemic Pre-Conditioning or Hemin Treatment
Source: PLoS One. 2012 Nov 14;7(11):e49569. doi: 10.1371/journal.pone.0049569 (PMC3498198; doi:10.1371/journal.pone.0049569)
Supplement: Table S11 — Down regulated genes in IPC group (vs control), according to GO and KEGG categories. (DOC) [file pone.0049569.s011.doc]

**Table S11.** Down regulated genes in IPC group (vs control), according to GO and KEGG categories.

| **CATEGORIES** | **Differentially expressed genes** |
| --- | --- |
| **Olfactory Transduction** | Olfr17, Olfr684, Olfr12, Olfr1350, Olfr1276, Olfr1215, Olfr971, Olfr441, Olfr1459, Olfr478, Olfr661, Olfr608, Olfr1406, Olfr906, Olfr620, Olfr975, Olfr1126, Olfr161, Olfr1395, Olfr1280, Olfr1226, Olfr691, Olfr635, Olfr1371, Olfr1198 |

Differentially down-regulated genes after ischemic preconditioning and ischemia-reperfusion injury (IPC+IRI *vs* Control) classified in the most relevant GO and KEGG categories.
