# Supplementary material for: Transcriptome Analysis of Renal Ischemia/Reperfusion Injury and Its Modulation by Ischemic Pre-Conditioning or Hemin Treatment
Source: PLoS One. 2012 Nov 14;7(11):e49569. doi: 10.1371/journal.pone.0049569 (PMC3498198; doi:10.1371/journal.pone.0049569)
Supplement: Table S12 — Up regulated genes in Hemin+IRI group (vs IRI), according to GO and KEGG categories. (DOC) [file pone.0049569.s012.doc]

**Table S12.** Up regulated genes in Hemin+IRI group (vs IRI), according to GO and KEGG categories.

| **CATEGORIES** | **Differentially expressed genes** |
| --- | --- |
| **nervous system development** | Fgf18, Stmn1, Nedd4, Neurod2, Sema6c, Nrg1, Apob, Grip1 |
| **mitosis** | Hmga2, Nek1, Lmln, Spag5, 2810021B07Rik, Vcpip1, Csnk1a1 |
| **response to metal ion** | Fgb, Anxa7, Cdkn1b, Cp, Slc25a12, Fgg |
| **axonogenesis** | Actb, Dst, Stmn1, Ptpn11, Ptprz1 |
| **multicellular organism growth** | Gnas, Duox2, Gigyf2, Arid5b, Ankrd11 |
| **hormone-mediated signaling pathway** | Nr2f2, Ptpn11, Nr2c1, Nr2c2, Nr5a1 |
| **regulation of cell differentiation** | Bmpr2, Gnas, Lrp5, Nrg1, Apob |
| **cell projection assembly** | Prdx6, Nek1, Pcm1, Rock1, Pkhd1 |
| **vascular endothelial growth factor receptor signaling pathway** | Bmpr2, Fgf18, Nedd4, Myof |
| **regulation of blood pressure** | Bmpr2, Lrp5, Gucy1a3, Nisch |
| **regulation of cyclin-dependent protein kinase activity** | Nr2f2, Cdkn1b, Lmnb1, Ccnl1 |
| **RNA transport** | Tpr, Eif3a, Eif5b, Acin1, Eif3c, Upf3b |
| **vascular smooth muscle contraction** | Cyp4a14, Gnas, Plcb1, Rock1, Ppp1r12b, Gucy1a3 |
| **Wnt signaling pathway** | Lrp5, Plcb1, Rock1, Csnk1e, Chd8, Csnk1a1 |
| **arachidonic acid metabolism** | Cbr3, Cyp2b13, Cyp2b9, Cyp4a14, Ptges2 |
| **Salivary secretion** | Atp2b2, Gnas, Plcb1, Gucy1a3 |
| **endocrine and other factor-regulated calcium reabsorption** | Dnm3, Gnas, Plcb1 |
| **tryptophan metabolism** | Ddc, Kynu, Abp1 |
| **phenylalanine metabolism** | Prdx6, Ddc |

Differentially up-regulated genes after ischemia/ reperfusion injury in animals pre-treated with Hemin (IRI + Hemin x IRI), classified in the most relevant GO and KEGG categories.
