# Supplementary material for: Transcriptome Analysis of Renal Ischemia/Reperfusion Injury and Its Modulation by Ischemic Pre-Conditioning or Hemin Treatment
Source: PLoS One. 2012 Nov 14;7(11):e49569. doi: 10.1371/journal.pone.0049569 (PMC3498198; doi:10.1371/journal.pone.0049569)
Supplement: Table S13 — Down regulated genes in Hemin+IRI group (vs IRI), according to GO and KEGG categories. (DOC) [file pone.0049569.s013.doc]

**Table S13.** Down regulated genes in Hemin+IRI group (vs IRI), according to GO and KEGG categories.

| **CATEGORIES** | **Differentially expressed genes** |
| --- | --- |
| **apoptosis** | Pawr, Ntn1, Sh3glb1 |
| **actin filament bundle assembly** | Pawr, Pfn1 |
| **regulation of cell proliferation** | Nr3c1, Ccl5 |
| **intracellular protein transport** | Kpna4, Sec23a |
| **actin cytoskeleton organization** | Pfn1, Fmnl1 |
| **response to hypoxia** | Slc8a1, Sod3 |
| **sodium ion transport** | Slc8a1, Slc4a7 |
| **Toll-like receptor signaling pathway** | Ccl5, Map2k6 |
| **Glycosphingolipid biosynthesis - ganglio series** | Slc33a1 |

Differentially down-regulated genes after ischemia/ reperfusion injury in animals pre-treated with Hemin (IRI + Hemin x IRI), classified in the most relevant GO and KEGG categories.
