# Supplementary material for: Transcriptome Analysis of Renal Ischemia/Reperfusion Injury and Its Modulation by Ischemic Pre-Conditioning or Hemin Treatment
Source: PLoS One. 2012 Nov 14;7(11):e49569. doi: 10.1371/journal.pone.0049569 (PMC3498198; doi:10.1371/journal.pone.0049569)
Supplement: Table S14 — Up regulated genes in Hemin group (vs control), according to GO and KEGG categories. (DOC) [file pone.0049569.s014.doc]

**Table S14.** Up regulated genes in Hemin group (vs control), according to GO and KEGG categories.

| **CATEGORIES** | **Differentially expressed genes** |
| --- | --- |
| **multicellular organismal development** | Tshz1, Bax, Dhh, Dnase2a, Eya2, Eya3, Grn, Hoxb9, Hoxd4, Stmn1, Lrp5, Gadd45b, Sema4c, Sema6c, Sim1, Sort1, Trp63, Zfp521, Arhgap24, Bzw2, Tssk4, Ntng1 |
| **cell cycle** | Ccnt1, Cdkn2d, Smc2, Hmgb1, Lmnb1, Lrp5, Tcf3, Tgfb2, Trp63, Mapk7, Nsl1, Spag5, Gmnn, Cdt1, Nup43 |
| **nervous system development** | Agrn, Bax, Hmgb1, Stmn1, Dcdc2a, Sema4c, Sema6c, Sim1, Apob, Ghrl, Bzw2, Phf10, Grip1, Ntng1 |
| **regulation of signal transduction** | Agrn, Slc7a3, Inhba, Inppl1, Mdfic, Pdgfa, Tgfb2, Trp63, Muc20, Myof, Zfyve9, Dusp7, Ghrl, Pde6h |
| **DNA repair** | Cdkn2d, Eya2, Eya3, Hmgb1, Pola1, Ung, C77370, Rpa3, Ercc8, Tonsl, Swi5 |
| **MAPKKK cascade** | Mdfic, Lmnb1, Gadd45b, Pdgfa, Sema4c, Tgfb2, Muc20, Dusp7, Cdon, Ghrl, Pde6h |
| **positive regulation of cell differentiation** | Hmgb1, Inhba, Lrp5, Neo1, Tcf3, Tgfb2, Tgif2, Apob, Cdon |
| **RNA splicing** | U2af1, Dhx9, Rbm39, Hnrnpa3, Fus, Cir1, Snrpd3, Hnrpll |
| **regulation of cell migration** | Hmgb1, P2ry2, Pdgfa, Tmsb4x, Tgfb2 |
| **Complement and coagulation cascades** | Tfpi, Vwf, Cpb2, Fgg |
| **Arginine and proline metabolism** | Arg2, Gls2, Abp1 |
| **Vitamin digestion and absorption** | Apoa4, Apob |
| **Nitrogen metabolism** | Car8, Gls2 |
| **D-Glutamine and D-glutamate metabolism** | Gls2 |

Differentially up-regulated genes after Hemin treatment (Hemin x Control) classified in the most relevant GO and KEGG categories.
