# Supplementary material for: Transcriptome Analysis of Renal Ischemia/Reperfusion Injury and Its Modulation by Ischemic Pre-Conditioning or Hemin Treatment
Source: PLoS One. 2012 Nov 14;7(11):e49569. doi: 10.1371/journal.pone.0049569 (PMC3498198; doi:10.1371/journal.pone.0049569)
Supplement: Table S15 — List of selected genes used for microarray results validation by qRT-PCR. (DOC) [file pone.0049569.s015.doc]

**Table S15.** List of selected genes used for microarray results validation by qRT-PCR.

| **Gene symbol** | **Gene name** | **Taqman probes** |
| --- | --- | --- |
| **Fosl1** | fos-related antigen 1 | Mm04207958_m1 |
| **Cxcl1** | chemokine (C-X-C motif) ligand 1 | Mm04207460_m1 |
| **Socs3** | suppressor of cytokine signaling 3 | Mm00545913_s1 |
| **Hmox1** | heme oxygenase (decycling) 1 | Mm00516004_m1 |
| **Ccl5** | chemokine (C-C motif) ligand 5 | Mm01302427_m1 |
| **Muc20** | mucin 20 | Mm00524818_m1 |
| **Hoxd4** | homeobox D4 | Mm01333847_g1 |
| **Hprt** | hypoxanthine-guanine phosphoribosyltransferase | Mm00446968_m1 |
